# Interleukin-6 Receptor Signaling and Abdominal Aortic Aneurysm Growth Rates

**DOI:** 10.1161/CIRCGEN.118.002413

**Published:** 2019-02-19

**Authors:** Ellie Paige, Marc Clément, Fabien Lareyre, Michael Sweeting, Juliette Raffort, Céline Grenier, Alison Finigan, James Harrison, James E. Peters, Benjamin B. Sun, Adam S. Butterworth, Seamus C. Harrison, Matthew J. Bown, Jes S. Lindholt, Stephen A. Badger, Iftikhar J. Kullo, Janet Powell, Paul E. Norman, D. Julian A. Scott, Marc A. Bailey, Stefan Rose-John, John Danesh, Daniel F. Freitag, Dirk S. Paul, Ziad Mallat

**Affiliations:** 1 National Centre for Epidemiology and Population Health, Research School of Population Health, The Australian National University, Canberra, Australia (E.P.).; 2 Division of Cardiovascular Medicine (M.C., F.L., J.R., C.G., A.F., J.H., Z.M.), University of Cambridge, United Kingdom.; 3 BHF Cardiovascular Epidemiology Unit, Department of Public Health and Primary Care (E.P., M.S., J.E.P., B.B.S., A.S.B., J.D., D.F.F., D.S.P.), University of Cambridge, United Kingdom.; 4 Université Côte d’Azur, Institut National de la Sante et de la Recherche Medicale, Centre Mediterranéen de Recherche Moleculaire (F.L., J.R.).; 5 University Hospital of Nice, France (F.L., J.R.).; 6 Department of Health Sciences (M.S.), University of Leicester.; 7 Department of Cardiovascular Sciences, NIHR Leicester Biomedical Research Centre (S.C.H., M.J.B.), University of Leicester.; 8 British Heart Foundation Centre of Excellence, Division of Cardiovascular Medicine, Addenbrooke’s Hospital, Cambridge, UK (J.E.P., A.S.B., S.C.H., J.D., D.F.F., D.S.P., Z.M.).; 9 NIHR Blood and Transplant Research Unit in Donor Health and Genomics, Cambridge, United Kingdom (A.S.B., J.D.).; 10 Department of Cardiovascular and Thoracic Surgery, Elitary Research Centre of Individualised Medicine in Arterial Disease, Odense University Hospital, Denmark (J.S.L.).; 11 Regional Vascular Surgery Unit, Belfast Health and Social Care Trust, United Kingdom (S.A.B.).; 12 Department of Cardiovascular Medicine, Gonda Vascular Center, Mayo Clinic, Rochester, MN (I.J.K.).; 13 Faculty of Medicine, Department of Surgery and Cancer, Imperial College London, United Kingdom (J.P.).; 14 Medical School, University of Western Australia, Perth, Australia (P.E.N.).; 15 Leeds Vascular Institute, Leeds General Infirmary (D.J.A.S., M.A.B.).; 16 Leeds Institute of Cardiovascular and Metabolic Medicine, School of Medicine, University of Leeds, United Kingdom (D.J.A.S., M.A.B.).; 17 Department of Biochemistry, Christian-Albrechts-University, Kiel, Germany (S.R.-J.).; 18 Department of Human Genetics, Wellcome Sanger Institute, Hinxton, United Kingdom (J.D.).; 19 Institut National de la Santé et de la Recherche Médicale, Paris Cardiovascular Research Center, France (Z.M.).

**Keywords:** alleles, aortic aneurysm, genetics, inflammation, interleukins

## Abstract

**Background:**

The Asp358Ala variant (rs2228145; A>C) in the IL (interleukin)-6 receptor (*IL6R*) gene has been implicated in the development of abdominal aortic aneurysms (AAAs), but its effect on AAA growth over time is not known. We aimed to investigate the clinical association between the *IL6R*-Asp358Ala variant and AAA growth and to assess the effect of blocking the IL-6 signaling pathway in mouse models of aortic aneurysm rupture or dissection.

**Methods:**

Using data from 2863 participants with AAA from 9 prospective cohorts, age- and sex-adjusted mixed-effects linear regression models were used to estimate the association between the *IL6R*-Asp358Ala variant and annual change in AAA diameter (mm/y). In a series of complementary randomized trials in mice, the effect of blocking the IL-6 signaling pathways was assessed on plasma biomarkers, systolic blood pressure, aneurysm diameter, and time to aortic rupture and death.

**Results:**

After adjusting for age and sex, baseline aneurysm size was 0.55 mm (95% CI, 0.13–0.98 mm) smaller per copy of the minor allele [C] of the Asp358Ala variant. Change in AAA growth was −0.06 mm per year (−0.18 to 0.06) per copy of the minor allele; a result that was not statistically significant. Although all available worldwide data were used, the genetic analyses were not powered for an effect size as small as that observed. In 2 mouse models of AAA, selective blockage of the IL-6 trans-signaling pathway, but not combined blockage of both, the classical and trans-signaling pathways, was associated with improved survival (*P*<0.05).

**Conclusions:**

Our proof-of-principle data are compatible with the concept that IL-6 trans-signaling is relevant to AAA growth, encouraging larger-scale evaluation of this hypothesis.

## Abstract 2

Supplemental Digital Content is available in the text.

Abdominal aortic aneurysms (AAAs) are defined as an enlargement of the aorta to ≥30 mm diameter. They usually grow asymptomatically until rupture occurs, after which the survival of affected individuals is <20%.^1^ AAAs typically occur in mid-to-later life and more commonly in men (prevalence of 1%–2%^2,3^ compared with <1% in women^4^). Current standard of care is surgical intervention, either open surgery or endovascular repair. However, because of surgical risks versus benefits, such interventions are generally recommended only for people with larger AAAs (diameter ≥55 mm or >40 mm and enlarging >10 mm/y).^5,6^ The growth rates of AAAs vary considerably between individuals^7^ and there is currently a lack of therapeutic options to slow or halt progression of AAAs.^8^ Inflammatory processes in the vessel wall may contribute to the progression of AAAs.^9–11^ For example, levels of circulating inflammatory markers including IL (interleukin)-6 are higher in prevalent AAA cases than controls^12^ and correlate with the size of AAA in cross-sectional studies.^13^

IL-6 is a central coordinator of inflammatory responses by controlling the systemic inflammatory response in the liver and the activation and differentiation of leukocyte subsets, including macrophages and T cells. IL-6 signaling occurs in 2 different modes, termed classical (cis-) and trans-signaling. In classical signaling, binding of IL-6 to the mIL-6R (membrane-bound IL-6 receptor) induces homodimerization with its coreceptor gp130, resulting in the phosphorylation of the transcription factors STAT3 and STAT1.^14,15^ Hence, classical signaling is dependent on the membrane-bound form of the IL-6 receptor and occurs only in leukocyte subsets and hepatocytes that express this molecule. By contrast, trans-signaling occurs through a circulating soluble form of IL-6R (sIL-6R), which, if bound to IL-6, is able to stimulate cells expressing gp130, even in the absence of mIL-6R.^14,16^ Because gp130 is almost ubiquitously expressed, IL-6 trans-signaling can occur in virtually any cell, although is probably active only during conditions of immunologic stress.^14,16^

A nonsynonymous variant (Asp358Ala; rs2228145 A>C) in the *IL6R* gene, encoding the IL-6 receptor, plays a critical role in IL-6 signaling. The minor allele [C] of this variant is associated with a reduced risk of several chronic conditions, including coronary heart disease,^17^ atrial fibrillation,^18^ rheumatoid arthritis,^19^ type 1 diabetes mellitus,^20^ but an increased risk of asthma.^21^ The variant results in more efficient proteolytic cleavage of mIL-6R, thereby reducing levels of mIL-6R and dampening classical signaling.^22,23^ Conversely, the variant increases levels of sIL-6R, although the exact effects of the variant on the trans-signaling pathway are unknown.^20^ A Mendelian randomization study has previously implicated the rs2228145 in the causal pathway of AAA, with the minor allele [C] showing a protective effect for the risk of AAA and a combined end point of rupture or surgical intervention.^12^ Licenced drugs are available that target the IL-6/IL-6 receptor pathway. However, evidence is needed that this pathway is associated with aneurysm progression or rupture to encourage repurposing drugs for use in patients with known AAA.

The aims of this study were (1) to assess and quantify the effect of the functional *IL6R* variant on the progression of AAAs in population cohorts with prospective follow-up and standardized repeated measurements of AAA diameter and (2) to estimate the effect of blocking the IL-6 signaling pathway (ie, either both classical and trans-signaling pathways or specifically the trans-signaling pathway) on time to aneurysm rupture in mouse models.

## Methods

Details on the materials and methods are available in the Data Supplement. Because of the sensitive nature of the data collected for this study, requests to access the human AAA datasets from qualified researchers trained in human subject confidentiality protocols may be sent to the study leaders of each cohort. The experimental data that support the findings of this study are available from the corresponding author on reasonable request. Each human AAA cohort was approved by a research ethics committee and all participants gave informed consent. Animal experiments were approved by the UK Home Office and performed under PPL PA4BDF775. The care and use of all mice in this study was performed in accordance with the UK Home Office regulations under the Animals (Scientific Procedures) Act 1986.

## Results

### Association of *IL6R*-Asp358Ala With AAA Growth Rate

**Figure 1.**
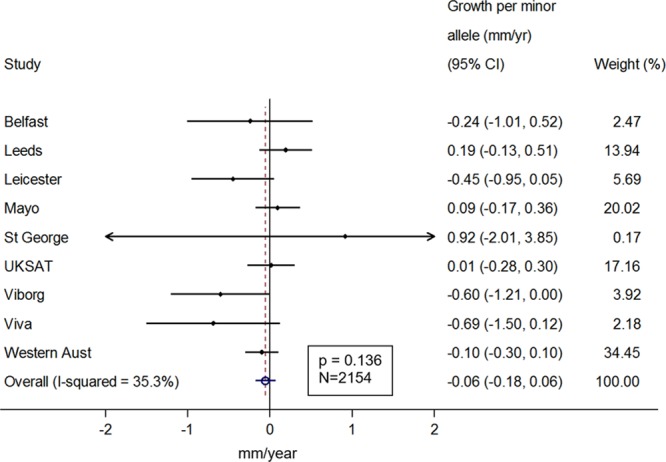
**Sex- and age-adjusted change in abdominal aortic aneurysm growth rate (mm/y) per copy of the IL (interleukin)-6 minor allele.**

We studied a total of 2863 participants across 9 prospective cohorts, 91% of whom were men (mean age of 72 years; Table 1). AAA size was on average 43 mm at baseline and participants had an average of 4 scans across an average of 3 years (Table 1). A summary of baseline characteristics by *IL6R*-rs2228145 (or proxy) genotype is given in Table I in the Data Supplement. On average, aneurysms grew by 1.88 mm per year (95% CI, 1.79–1.96; Figure I in the Data Supplement). Baseline aneurysm size was 0.55 mm (0.13–0.98) smaller per copy of the minor allele [C] (Figure II in the Data Supplement). Among those with at least 2 measurements of aneurysm size (n=2154), there was no statistically significant growth rate in AAA per minor allele (growth per minor allele =−0.06 mm/y [−0.18 to 0.06]; Figure 1). All association analyses were adjusted for age and sex. Results were similar after adjustment for current smoking status, diabetes mellitus status, body mass index, and aneurysm measurement method (Figure III in the Data Supplement).

**Table 1.**
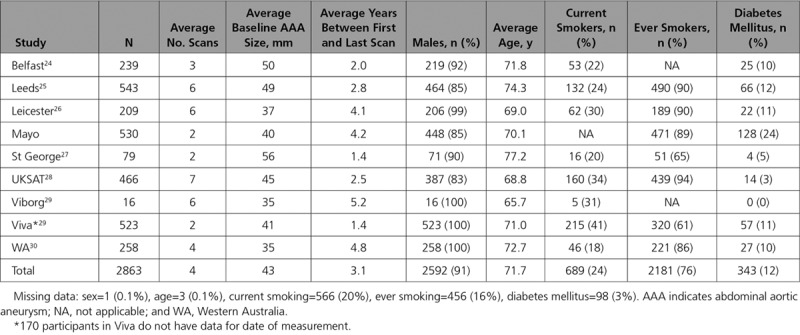
Baseline Characteristics of the Studies Included in the Human Genetic Analysis

Similar results were observed when the analysis was restricted to those with a small aneurysm at baseline (growth =−0.10 mm/y [−0.23 to 0.02] per copy of the minor allele; Figure IV in the Data Supplement) or medium aneurysm at baseline (growth =−0.08 mm/y [−0.21 to 0.05] per copy of the minor allele; Figure V in the Data Supplement). We did observe an association between the Asp358Ala variant and time to surgery threshold after adjusting for age and sex (hazards ratio =0.85 [0.73–0.98] per copy of the minor allele; Figure VIA in the Data Supplement). The hazards ratio was in the same direction but became statistically nonsignificant in the subset of studies for which we were able to additionally adjust for current smoking, diabetes mellitus status, body mass index, and measurement method (hazards ratio =0.91 [0.77–1.06]; Figure VIB in the Data Supplement). The overall change in AAA growth remained the same when individuals with only a single measure of aneurysm size were included in the model (n=2691, growth =−0.06 mm/y [−0.18 to 0.06]; Figure VII in the Data Supplement).

### Inhibition of IL-6 Signaling Pathway in Angiotensin II + Anti-TGF-β Mouse Model

**Figure 2.**
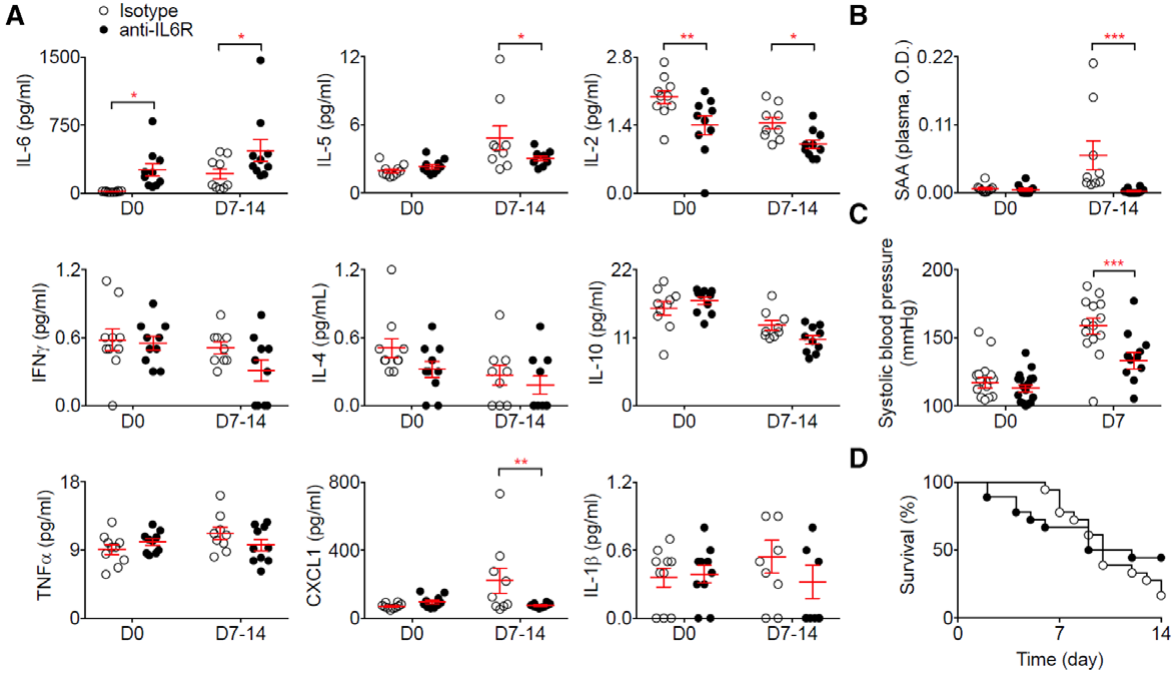
**Anti-IL (interleukin)-6R prevents Ang II (angiotensin II)–induced hypertension but does not protect against aortic rupture induced by Ang II and anti-TGF (transforming growth factor)-β infusion.** Mice were treated with anti-IL-6R or isotype control (n=22 mice/group) starting one week before Ang II and anti-TGF-β infusion. **A**, Plasma concentration of cytokines at day 0 (before Ang II and anti-TGF-β infusion) and day 7 to 14. *P<0.05 isotype vs anti-IL-6R; **P<0.01 isotype vs anti-IL-6R; 2-way ANOVA followed by uncorrected Fisher test. **B**, Plasma concentration of serum amyloid A (SAA) at day 0 (before Ang II and anti-TGF-β infusion) and day 7 to 14. ***P<0.05 isotype vs anti-IL-6R; 2-way ANOVA followed by uncorrected Fisher test. **C**, Systolic blood pressure measurement using tail cuff at day 0 and 7 after Ang II and anti-TGF-β infusion. ***P<0.001 isotype vs anti-IL-6R; 2-way ANOVA followed by uncorrected Fisher test. **D**, Survival curves of mice after Ang II and anti-TGF-β infusion. All data for the generation of the graphs shown in Figure 2 were generated in 2 independent experiments and then pooled together.

We next tested the effect of blocking the IL-6 pathway in 2 distinct, previously characterized mouse models of AAA (Methods). In the Ang II (angiotensin II) + anti-TGF (transforming growth factor)-β model, mice infused with anti-IL-6R (blocking both classical and trans-signaling pathways) demonstrated a significant increase of plasma concentration of IL-6, as compared to isotype-treated mice, and this difference was sustained over the course of the experiment (Figure 2A). We observed a reduction in plasma concentrations of serum amyloid A, a protein expressed in response to inflammation, after blocking IL-6R compared with the control mice (Figure 2B). Blocking IL-6R significantly reduced plasma concentration of IL-2 before and after the infusion and reduced concentration of IL-5 and CXCL1 (chemokine ligand 1) after the infusion (Figure 2A). After anti-IL-6R treatment, systolic blood pressure was significantly lower after the infusion compared with control treatment (Figure 2C). However, there was no significant difference in rate of aneurysm rupture between the anti-IL-6R treated and control groups (Figure 2D). Because there was no observed association with AAA rupture, we did not further assess the effect of blocking the IL-6R pathway on AAA growth.

**Figure 3.**
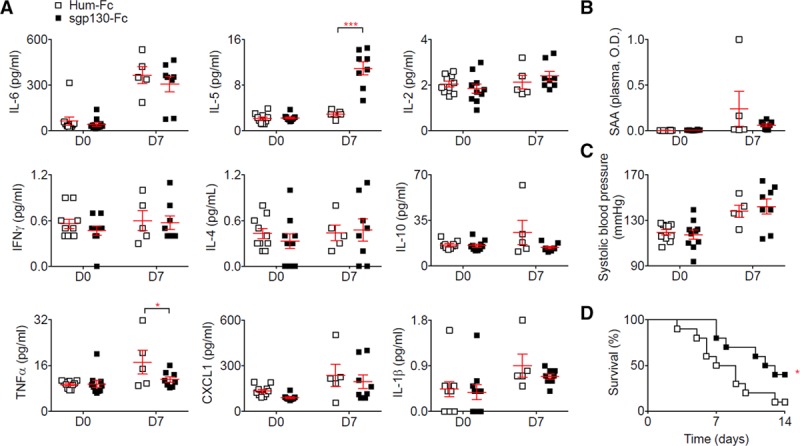
**Selective blockage of the IL (interleukin)-6 trans-signaling pathway using sgp130 in the Ang II (angiotensin II) + anti-TGF (transforming growth factor)-β model reduces aortic rupture.** Mice were treated with sgp130Fc or human IgG1-Fc (Hum-Fc; n=10 mice/group) starting one week before Ang II and anti-TGF-β infusion. **A**, Plasma concentration of cytokines at day 0 (before Ang II and anti-TGF-β infusion) and day 7. *P<0.05 Hum-Fc vs sgp130; ***P<0.001 Hum-Fc vs sgp130; 2-way ANOVA followed by uncorrected Fisher test. **B**, Plasma concentration of serum amyloid A (SAA) at day 0 (before Ang II and anti-TGF-β infusion) and day 7. **C**, Systolic blood pressure measurement using tail cuff at day 0 and day 7 after Ang II and anti-TGF-β infusion. **D**, Survival curves of mice after Ang II and anti-TGF-β infusion. *P<0.05 Hum-Fc vs sgp130; Gehan-Breslow-Wilcoxon test. All data for the generation of the graphs shown in Figure 3 were generated in one independent experiment.

Selectively blocking the IL-6 trans-signaling pathway using sgp130Fc did not change the concentration of IL-6 (Figure 3A) or serum amyloid A (Figure 3B), but significantly induced IL-5 and reduced TNF (tumor necrosis factor)-α plasma concentration (Figure 3A). Although we observed no difference in systolic blood pressure between the mice treated with sgp130Fc and the control mice (Figure 3C), there was a significant reduction in aneurysm rupture after sgp130Fc treatment compared with control treatment (Figure 3D).

### Inhibition of IL-6 Signaling Pathway in Elastase + Anti-TGF-β Mouse Model

**Figure 4.**
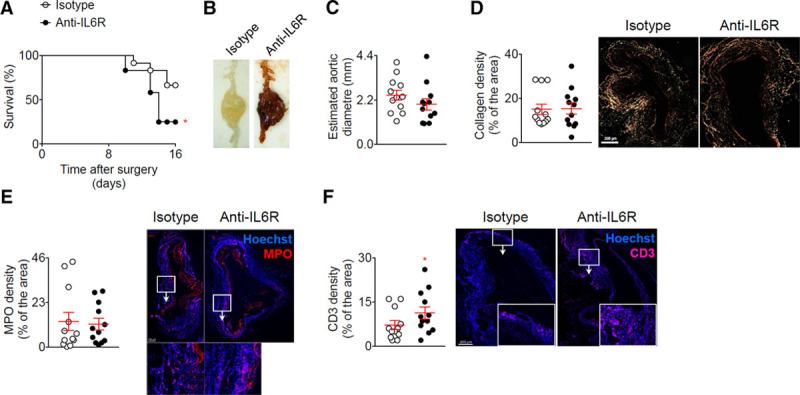
**Blockage of the IL (interleukin)-6 pathway using anti-IL-6R in the elastase + anti-TGF (transforming growth factor)-β model enhances T-cell infiltration and rupture of the aorta.** Mice were treated with anti-IL-6R or isotype control (n=12 mice/group) starting one week before the application of elastase and the infusion anti-TGF-β. **A**, Survival curves of mice after the application of elastase and the infusion anti-TGF-β. *P<0.05 isotype vs anti-IL-6R; Gehan-Breslow-Wilcoxon test. **B**, Representative macroscopic pictures of abdominal aortic aneurysms from mice treated with elastase and anti-TGF-β and isotope or anti-IL-6R, at day 16. Note that the aneurysm from the isotype treated mouse was not ruptured. **C**, Analysis of the aortic diameter (µm) based on the perimeter obtained from aortic cross sections. **D**, Quantification and representative images of collagen content of the aortic wall analyzed using Sirius Red staining under polarized light. **E** and **F**, Quantification and representative images of myeloperoxidase (MPO) (**D**) and CD3 (**E**) immunofluorescent stainings on aortic cross section. *P<0.05 isotype vs anti-IL-6R; Mann-Whitney test. All data for the generation of the graphs shown in Figure 4 were generated in one independent experiment.

Using the elastase + anti-TGF-β model, we found that blockage of the IL-6R pathway using anti-IL-6R resulted in significantly increased mortality (Figure 4A) induced by aortic rupture (Figure 4B) but there was no change in the diameter of the aneurysm at the end of the experiment (Figure 4C). α-smooth muscle actin (α-SMA^+^) density in the media was similar in the 2 groups of mice (data not shown). There was also no change in the collagen content of the aortic wall (Figure 4D) or the recruitment of myeloperoxidase positive (MPO^+^) cells (Figure 4E), but treatment with anti-IL-6R significantly enhanced the recruitment of CD3^+^ T cells in the aortic wall (Figure 4F).

**Figure 5.**
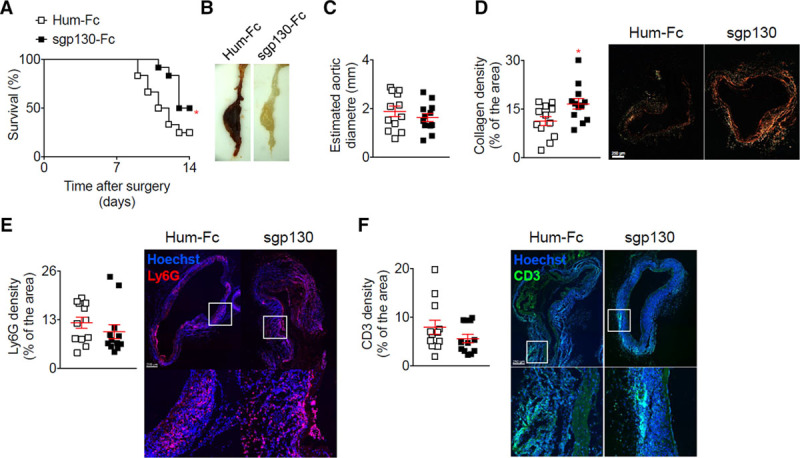
**Selective blockage of the IL (interleukin)-6 trans-signaling pathway using sgp130 in the elastase + anti-TGF (transforming growth factor)-β model increases collagen deposition and prevents aortic rupture.** Mice were treated with sgp130 or Hum-Fc (n=12 mice/group) starting on the day of the application of elastase and the infusion anti-TGF-β. **A**, Survival curves of mice after the application of elastase and the infusion anti-TGF-β. *P<0.05 Hum-Fc vs sgp130; Gehan-Breslow-Wilcoxon test. **B**, Representative macroscopic pictures of abdominal aortic aneurysms from mice treated with elastase and anti-TGF-β and isotope or anti-IL-6R, at day 16. Note that the aneurysm from the Hum-Fc treated mouse was ruptured. **C**, Analysis of the aortic diameter (µm) based on the perimeter obtained from aortic cross sections. **D**, Quantification and representative images of collagen content of the aortic wall analyzed using Sirius Red staining under polarized light. *P<0.05 Hum-Fc vs sgp130; Mann-Whitney test. **E** and **F**, Quantification and representative images of lymphocyte antigen 6 complex, locus G (Ly6G) (**D**) and cluster of differentiation 3 (CD3) (**E**) immunofluorescent stainings on aortic cross-section. All data for the generation of the graphs shown in Figure 5 were generated in one independent experiment.

Blocking only the IL-6 trans-signaling pathway using sgp130Fc significantly increased survival (Figure 5A) by reducing aortic ruptures (Figure 5B), although at the end of the experiment there was no change in the aortic diameter between the treated and control mice (Figure 5C). Histological analysis of aortic samples revealed a significant increase in the collagen content of the arterial wall (Figure 5D) but no differences in α-SMA^+^ density (data not shown), Ly6G^+^ (Figure 5E) and CD3^+^ T-cell accumulation (Figure 5F) after sgp130Fc infusion, as compared to the control mice. Table 2 summarises the results of the different mouse models with a comparison to the human genetic data.

**Table 2.**
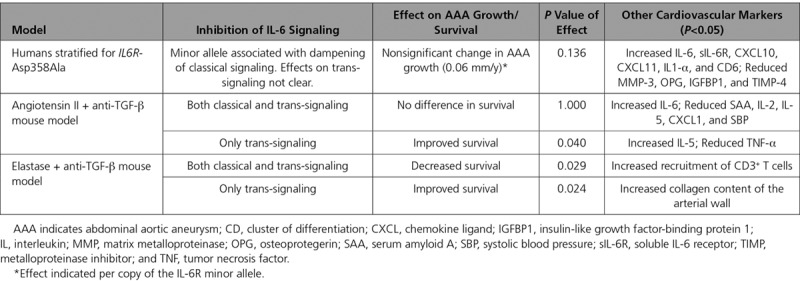
Comparison of Results for the Association Between IL-6R and Abdominal Aortic Aneurysm From Human Genetic Analysis and Mouse Experimental Models

### Evaluation of the Effect of *IL6R*-rs2228145 on a Range of Cardiovascular Markers

As would be expected, the minor allele of *IL6R*-rs2228145 was associated with increased plasma concentrations of IL-6 and sIL-6R. The variant was also associated with increased monocyte count, after correcting for multiple comparisons (*P*<1.389×10^−3^). At a nominal significance level (*P*<0.05), rs2228145-C was associated with reduced lymphocyte count, increased levels of the cytokines CXCL10, CXCL11, and IL1-α, as well as CD6 (an important regulator of T cells), and reduced levels of MMP-3 (a matrix metalloproteinase), TIMP-4 (a metalloproteinase inhibitor), OPG (osteoprotegerin), and IGFBP1 (Figure VIII in the Data Supplement). In our analysis, the effects on plasma IL-2, IL-5, and CXCL1 levels, as well as on blood pressure, were not statistically significant.

## Discussion

In a combined analysis of the available worldwide clinical genetic data on AAA growth, we observed no statistically significant decrease in annual AAA growth rates for carriers of the minor allele of the Asp358Ala variant (rs2228145) in the *IL6R* gene. While we did observe a 15% decrease in the rate of reaching the surgery threshold of ≥55 mm (hazards ratio =0.85 [0.73–0.98] per copy of the minor allele), people with copies of the *IL6R*-Asp358Ala variant also had, on average, smaller baseline aneurysm diameters. Although we tried to account for this by allowing baseline hazards to vary depending on initial aneurysm size, some residual confounding is possible and could explain the observed results. In experimental data from mouse models, we found that selective blockade of the IL-6 trans-signaling pathway was associated with decreased aortic rupture and death. In exploratory analyses of cardiovascular and inflammatory biomarkers in healthy participants, we found that rs2228145-C was inversely associated with plasma levels of OPG, MMP-3, and TIMP-4 (*P*<0.05). OPG has previously been shown to promote MMP release from monocytes and vascular smooth muscle cells,^31,32^ and aberrant aortic ECM (extracellular matrix) remodeling has been suggested to play a key role in the pathogenesis of AAA.^33^ However, we note that further studies are needed to validate our biomarker data. Taken together, these human genetic, biomarker, and experimental murine findings are compatible with the concept that IL-6 trans-signaling is relevant to AAA growth, encouraging larger-scale evaluation of this hypothesis.

**Figure 6.**
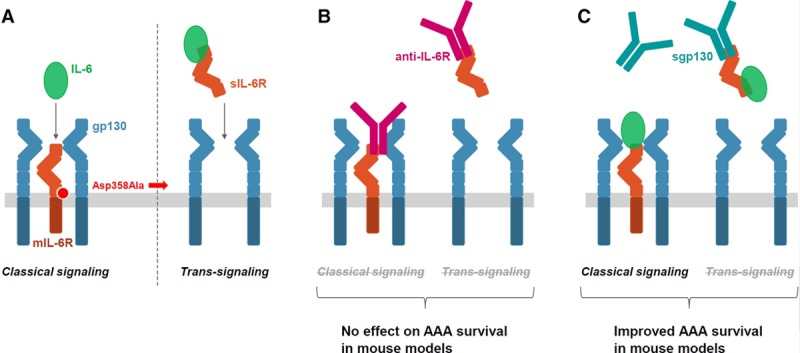
**Overview of the IL (interleukin)-6 classical and trans-signaling pathways and their potential role in abdominal aortic aneurysm (AAA) growth. A**, In classical IL-6 signaling (**left**), the binding of the cytokine IL-6 to the mIL-6R (membrane-bound IL-6 receptor) leads to the dimerization of its coreceptor gp130, and subsequently, triggers downstream signaling in a restricted subset of cells. In IL-6 trans-signaling (**right**), IL-6 forms a complex with sIL-6R (soluble IL-6 receptor) that can stimulate cells expressing gp130 even in the absence of mIL-6R. The minor allele of a functional variant in the *IL6R* gene, Asp358Ala (rs2228145 A>C) results in more efficient proteolytic cleavage of mIL-6R, thereby reducing levels of mIL-6R and classical IL-6 signaling but potentially increasing trans-signaling. **B**, In mouse models of AAA, the blockage of both the classical and trans-signaling pathways with anti-IL-6R (ie, MR16-1, the animal-equivalent of tocilizumab) did not have a conclusive effect on the time to aneurysm rupture. **C**, Specific blockage of the trans-signaling pathway with sgp130 resulted in improved survival rates in mouse models of AAA.

If increased availability of sIL-6R results in a dampening of the IL-6 trans-signaling pathway, this may explain potential protective effects in AAA and is consistent with previously observed protective effects in mouse models of sepsis^34^ and pancreatic and lung failure.^35^ As we found a consistent pattern of results when the trans-signaling pathway was selectively blocked in the mouse models, it suggests that this pathway could have a detrimental effect on AAA growth (Figure 6). For example, the minor allele of the rs2228145 variant may result in a local reduction of IL-6 trans-signaling in the abdominal vasculature, reducing AAA risk^12^ and, perhaps, AAA growth rates. Our observation of only a small, but statistically insignificant, decrease in annual AAA growth rates does not preclude meaningful clinical effects, because the growth rate reduction estimated from natural genetic variation does not necessarily relate to the magnitude of the benefit that might result from pharmacological treatment directed at the IL-6 trans-signaling pathway.^36^ Selective blocking of the IL-6 trans-signaling pathway using sgp130Fc is being investigated in phase II clinical trials in patients with inflammatory bowel disease.^14^

Nevertheless, apparent inconsistencies in our findings require further elucidation. For example, blockade of both the classical and trans-signaling IL-6 pathways using the animal-equivalent (MR16-1) of tocilizumab had no effect on AAA rupture in the Ang II + anti-TGF-β model, but it was associated with decreased survival in the elastase + anti-TGF-β model. These different outcomes could be explained by differences in the development of AAA in the different mouse models. The primary process of aneurysm formation in the angiotensin model is a medial dissection, which may be accentuated by elevations in blood pressure (even though high blood pressure is not the primary cause of medial dissection). Hence, the potential protection afforded by the MR16-1 antibody in this model can at least in part be attributed to the significant reduction of blood pressure. In contrast, the elastase model does not involve medial dissection or elevations in blood pressure, but induces progressive remodeling, dilatation, and eventually transmural rupture of the artery wall, better mimicking AAA progression in humans.^37,38^

If selective blockade of the IL-6 trans-signaling pathway results in decreased aortic rupture, as suggested by our murine data, one might expect that blocking both the classical and trans-signaling pathways would also result in decreased aortic rupture. However, we did not observe such a finding, perhaps because of competing downstream actions of the classical and trans-signaling pathways. Such an explanation is consistent with our finding that IL-5 levels were increased and TNF-α levels decreased when trans-signaling was selectively blocked, whereas blockade of both classical and trans-signaling pathways led to reductions in IL-5 levels and no changes in TNF-α. Our findings suggest that selective blockade of the IL-6 trans-signaling pathway, compared to blockade of both IL-6 signaling pathways, results in different downstream cytokine profiles and potentially different effects on AAA progression. It is also possible that the blocking of both the classical signaling cascade (considered to have protective and regenerative cellular effects) and trans-signaling cascade (considered to have proinflammatory effects) canceled each other out, leading to no detectable effect on AAA rupture. Further studies are needed to replicate and further characterize our findings.

We undertook a range of sensitivity analyses to test assumptions underlying our longitudinal human genetic studies. We studied complementary murine models of AAA, including the elastase + anti-TGF-β mouse model that has been shown to more closely mimic the AAA growth and rupture patterns seen in humans. To generate new mechanistic hypotheses, we conducted exploratory studies of the *IL6R*-Asp358Ala variant in relation to cardiovascular and inflammatory plasma biomarkers recorded in healthy participants. The experimental mouse studies were conducted under severe conditions in which TGF-β was blocked. Although any effect of IL-6R signaling might have been easier to observe in a less severe model (aortic dilatation without rupture), a protective effect of the intervention in that setting would not provide assurance that the intervention will also be protective in a more severe model (aortic rupture). A treatment that limits aortic dilatation but does not reduce the risk of aortic rupture would have limited clinical relevance.

Our study had potential limitations. It was powered to detect reductions in aneurysm growth of ≈0.21 mm per year or larger, much greater than the observed nonsignificant decrease of 0.06 mm per year. Future studies powered to see an effect the same size as that observed in the current study would need to recruit an additional ≈21 500 participants (total participants needed=24 444, Material in the Data Supplement). This is unlikely to be achievable in the near future; alternative study methods using a composite phenotype for disease progression may be needed. Index event and survival bias, in which participants are selected into the study based on both having and surviving an event, may have biased the results towards the null.^39^ However, this bias is likely to be small (<10%).^39^ Further, ultrasound, the primary method used to assess AAA diameter in the included studies, has a margin of error of 2 to 3 mm,^40^ greater than the annual rate of aneurysm growth, making changes in growth difficult to detect. This might be why we observed an association between the *IL6R*-Asp358Ala variant and time to surgery threshold of ≥55 mm but not when looking at continuous change in AAA size. Although we examined rupture rather than aortic diameter as the outcome in the mouse experimental models, our published data indicate that aortas that rupture have larger diameters or faster diameter progression than the ones that do not rupture.^30^

It is also uncertain how well the results of our animal models translate to clinical disease. For example, an important difference is that IL-6 blockage is initiated before or at the time of disease development in the mice models of AAA, thereby not truly mimicking the treatment effects expected in humans, in which drugs to block IL-6 pathway would be started after disease onset. Blockade of the IL-6 signaling pathways in the Ang II + anti-TGF-β mouse model resulted in reproducible reductions in systolic blood pressure. Although tocilizumab has been anecdotally reported to improve pulmonary hypertension in Castleman disease,^41,42^ the rs2228145 variant was not associated with changes in systemic blood pressure in healthy participants in a genome-wide association study. A large-scale randomized trial found no difference in the number of hypertension events reported in those using tocilizumab compared to placebo.^43,44^ Thus, the acute responses to pharmacological doses of Ang II in the mouse model may not faithfully reproduce the human setting of AAA.

Our study may have clinical implications. Tocilizumab is currently indicated in a few disease settings, including rheumatoid arthritis and giant cell arteritis, both of which are associated with an increased risk of aortic aneurysm.^45,46^ The development of coronary artery aneurysms has also been reported in a nonplacebo-controlled pilot study of tocilizumab in children with Kawasaki Disease.^47^ Findings from this pilot study in children, combined with our finding that blocking the IL-6 pathway using the animal-equivalent of tocilizumab was associated with decreased survival in the elastase + anti-TGF-β model, suggests that patients treated with tocilizumab for conditions associated with aortic aneurysm development should possibly be monitored for AAA.

In conclusion, our proof-of-principle data are potentially compatible with the concept that IL-6 trans-signaling is relevant to AAA growth, encouraging larger-scale evaluation of this hypothesis.

## Acknowledgments

We thank Chugai Pharmaceutical Co, Ltd for providing the MR16-1. We thank Tao Jiang and Praveen Surendran (Cardiovascular Epidemiology Unit, University of Cambridge) for providing access to genomic datasets.

## Sources of Funding

The Cardiovascular Epidemiology Unit is supported by the UK Medical Research Council (MR/L003120/1), British Heart Foundation (RG/13/13/30194), and National Institute for Health Research (Cambridge Biomedical Research Centre at the Cambridge University Hospitals NHS Foundation Trust). The views expressed are those of the authors and not necessarily those of the NHS, the NIHR or the Department of Health and Social Care. Dr Rose-John was supported by grants of the Deutsche Forschungsgemeinschaft (CRC877, project A1) and the German Cluster of Excellence 306 Inflammation at Interfaces. The Leeds Aneurysm Development Study was funded by the Garfield Weston Foundation and Dr Bailey is supported by the British Heart Foundation. Dr Mallat is supported by the British Heart Foundation (RG/79120 and RG/79915).

## Disclosures

Dr Freitag has been a full-time employee of Bayer AG, Germany, since October 2015. The other authors report no conflicts.

## Supplementary Material
