# Supplementary material for: Interleukin-6 Receptor Signaling and Abdominal Aortic Aneurysm Growth Rates
Source: Circ Genom Precis Med. 2019 Feb 19;12(2):e002413. doi: 10.1161/CIRCGEN.118.002413 (PMC6383754; doi:10.1161/CIRCGEN.118.002413)
Supplement: Supplementary file 1 [file hcg-12-e002413-s001.pdf]

Supplemental Material

Contents

Detailed materials and methods....................2

Supplemental Data 1: List of RESCAN collaborators....................6

Supplemental Data 2: List of Aneurysm Consortium collaborators....................7

Supplemental Data 3: Power and sample size calculations.................... 8

Supplementary Table 1: Summary of baseline characteristics by IL6R genotype.................... 9

Supplementary Figure 1: Sex- and age-adjusted rate of change in AAA diameter....................10

Supplementary Figure 2: Sex- and age-adjusted difference in baseline AAA size per copy of the IL6R-rs2228145 minor allele....................10

Supplementary Figure 3: Change in AAA growth rate (mm/year) per copy of the IL6R-rs2228145 minor allele, after adjustment for age, sex, current smoking, diabetes status, body mass index and measurement method.................... 11

Supplementary Figure 4: Sex- and age-adjusted change in AAA growth rate (mm/year) per copy of the IL6R-rs2228145 minor allele, among people with small baseline size AAA.................... 11

Supplementary Figure 5: Sex- and age-adjusted change in AAA growth rate (mm/year) per copy of the IL6R-rs2228145 minor allele, among people with medium baseline size AAA.................... 12

Supplementary Figure 6a: Sex- and age-adjusted hazards ratios (HR) for time to surgery threshold (AAA size≥55mm) per copy of the IL6R-rs2228145 minor allele.................... 12

Supplementary Figure 6b: Hazards ratios (HR) for time to surgery threshold (AAA size≥55mm) per copy of the IL6R-rs2228145 minor allele, adjusted for age, sex, current smoking, diabetes status, body mass index and measurement method.................... 13

Supplementary Figure 7: Sex- and age-adjusted change in abdominal aortic aneurysm growth rate (mm/year) per copy of the IL6R-rs2228145 minor allele, including those single AAA measures.....13

Supplementary Figure 8: Associations between the IL6R-rs2228145 variant and intermediate traits and cytokine profiles.................... 14

References.................... 15

Detailed materials and methods

Human genetic analyses

Study participants. We analysed previously collected data from six prospective cohorts contributing to the RESCAN collaboration and three others (Belfast, Mayo Clinic [New York] and St George's [London]), that had repeated measurements of aneurysm diameter over time and genotyped data either for the *IL6R*-Asp358Ala variant (rs2228145) or for one of two proxy variants (rs4129267 or rs7529229; both r^2=1.0 with rs2228145, 1000 Genomes project). Genotype data for these cohorts was provided by the Aneurysm Consortium. Details on the RESCAN collaboration have been previously published and a list of RESCAN and Aneurysm Consortium collaborators is provided in Supplemental data 1 and 2. AAA was defined as having an aorta size ≥30mm.[1] Aneurysm diameter were measured by ultrasound, CT scan or MRI. Only participants that met the definition for AAA and had at least two diameter measurements on different dates were included in the current study.

Statistical analyses. Age- and sex-adjusted mixed-effects linear regression models were used to first model the change in AAA growth over time and then to estimate the association between rs2228145 (or the proxy variants rs4129267 or rs7529229) and baseline AAA size and annual change in AAA diameter (mm/year; estimated using interaction terms between time and copy of the minor allele). The main analysis was pre-specified to test the hypothesis that rs2228145 is associated with a decreased rate of AAA growth. Effect estimates and standard errors were pooled using inverse-variance weighted fixed-effects meta-analysis. 95% confidence intervals (CIs) were calculated for all effect sizes and an overall p-value for the pooled effect was calculated. Significance tests were two-sided. Heterogeneity between studies was quantified using the I^2 statistic.[2] To test whether adjustment for additional potential confounders changed the effect size, we re-ran the main analysis in a sub-sample of six studies with recorded data on current smoking, diabetes status, body mass index and method used to measure aneurysm size (i.e., ultrasound, CT scan, MRI or not specified), adjusting for these additional covariates.

Sensitivity analyses. Four sensitivity analyses were conducted. First, since participants with initially large aneurysms (i.e., those above the surgery threshold of 55mm) might be categorically different to participants with smaller aneurysms,[3] we re-ran the main model examining annual change in AAA diameter, and restricted the analysis to participants with a small baseline aneurysm diameter of 30-44mm. Aneurysms that grew larger than 45mm were censored in the analysis after the first measurement ≥45mm. Second, we also re-ran the main model restricting the analysis to participants with a medium baseline aneurysm diameter of 45-54 mm. Aneurysms that grew larger than 55mm were censored in the analysis after the first measurement ≥55mm. Third, a Cox proportional hazards model was used to examine the association between the rs2228145 variant (or proxy variant) and the rate of reaching the surgery threshold of ≥55mm, with baseline hazards estimated within 5mm baseline aneurysm size groups. Fourth, to increase statistical power, we re-ran the main analysis including participants with a single diameter AAA measure. The sensitivity analyses were exploratory and not pre-specified.

Power calculations for the main analysis are provided in Supplemental data 3. All analyses were done using Stata 13.1.

Phenome-scan. To explore the mechanisms through which inhibition of IL6-R signalling could influence AAA, we performed a genetic association analysis of the rs2228145 variant with a large panel of potentially relevant intermediate traits and cytokine profiles in healthy participants across several human genetic studies. We collected genetic association data for 36 white cell, red cell, and platelet properties in up 173,039 European-ancestry individuals;[4] systolic/ diastolic blood pressure and pulse pressure in up to 161,871 individuals;[5] and a wide range of plasma proteins in up to 4,981 individuals.[6]

Experimental mouse models

Mouse models. Several mouse models have been established that reproduce certain characteristics of aortic aneurysm development in humans, including inflammation, destruction of the extracellular matrix and aortic dilatation, but all have limitations with regards to the pathological interpretation of human AAA.[7] We tested the effect of blocking the IL-6 pathway on aortic rupture using two distinct experimental mouse models to study the role of IL-6 signalling in dissecting and non-dissecting AAAs. To investigate dissecting AAAs, mice were treated with a systematic infusion of angiotensin II with pharmacological inhibition of transforming growth factor-β (TGFβ) activity.[7] To examine non-dissecting AAAs, elastase was applied on top of the aorta and TGFβ activity was inhibited pharmacologically.[8] The angiotensin II and elastase models are widely used models for AAA, which are characterised by frequent ruptures and high reproducibility.[7] Blockage of the activity of TGFβ, a critical factor that maintains aortic wall integrity, exacerbates aneurysmal aortic dilatation, induces intraluminal thrombus formation and promotes aortic rupture, thereby reproducing the main features of human AAA.[8] Details on the development and characterisation of these mouse models have been previously published.[7, 8]

Procedures. For all experiments, we used male, C57Bl/6J, 8-week-old mice (Charles River, UK). Mice were infused with angiotensin II (Sigma) at a rate of 1µg/min/kg using osmotic minipumps (model 2004; ALZET). Alternatively, mice underwent surgery for the application of elastase (E1250; Sigma) at 10µl of the filtered solution for 3 minutes on top of the infrarenal aorta. Mice were injected intraperitoneally with anti-TGFβ (clone 1D11; BioXcell) using 250 µg/mouse at 3 times/week, starting on the day of the surgery (minipump implantation or application of elastase). All experiments were ended when the mortality reached 80%. Necropsies were performed to confirm the aortic rupture (thoracic or retroperitoneal hematoma) and aortic tissue samples were harvested, fixed in PFA 4 % overnight at 4°C, and stored in PBS at 4°C until further investigations. The analysis of the tissue sample was performed on all the animals, regardless of the presence of a rupture.
In both mouse models, mice were injected intravenously with a first bolus of 2mg of anti-IL6-R (clone MR16-1[9]; Chugai Pharmaceutical) or control isotype (clone HPRN; Bioxcell) one week before the experiment in order to induce immune tolerance to the antibody.[10] After surgery, mice were injected intraperitoneally with 500µg of anti-IL-6R twice a week to completely block the IL-6 pathway (i.e. inhibiting both classical and trans-signalling pathways) or with 500µg of a control isotype twice a week. We measured IL-6 concentration in the plasma of mice to confirm that the IL-6 pathway was blocked in the mice treated with

anti-IL-6R. In a separate experiment, the IL-6 trans-signalling pathway was selectively blocked using the fusion protein sgp130Fc[11] (supplied by Prof Stefan Rose-John, Institute of Biochemistry, Christian-Albrechts-University of Kiel, Germany). The mice received 10µg of sgp130Fc or control isotype (human IgG1 Fc; Bioxcell) three times a week starting from the first day of the experiment.

Phenotyping. Outcome measures included plasma levels of different biomarkers, systolic blood pressure, recruitment of T cells (CD3 staining) and neutrophils (MPO or Ly6G staining) to the aortic wall, collagen content of the aortic wall (Sirius red staining), aneurysm diameter, and time to aortic rupture and death.

Blood pressure measurements. Non-invasive blood pressure monitoring using tail cuff was done using a BP-2000 Blood Pressure Analysis System [Visitech Systems]. Mice were trained for blood pressure 2 days prior to taking the readings (10 readings/mouse). Blood pressure readings were taken before insertion of osmotic pumps and later at day 7 during the study.

Cytokine titration. For cytokine measurement, blood samples were obtained at day 0 and day 7 by saphenous bleed and at day 14 by intracardiac puncture. Blood was collected on EDTA, and plasma was obtained after centrifugation for 15 min at 12,000g and 4°C. Cytokine were measured using a V-PLEX Proinflammatory Panel 1 Mouse Kit [MSD] consisting of the following cytokines: IFN-γ, IL-10, IL-12p70, IL-1β, IL-2, IL-4, IL-5, IL-6,
KC/GRO and TNF-α. These measurements were conducted by the Core Biochemical Assay Laboratory at the Addenbrooke's Hospital, Cambridge, UK. Plasmatic concentration of SAA was analysed by ELISA [KMA0021, Thermo Fisher Scientific] according to the manufacturer's instructions.

Histology. Aortic tissues were cleaned of the surrounding tissues. Suprarenal (Angiotensin II model) or infrarenal (elastase model) aortic segments were incubated in 30% sucrose for 6-8 hours before inclusion in optical coherence tomography. Sections were stored at -80°C. For immunofluorescent stainings, sections were air dried, extensively washed in PBS and then permeabilized (0.1% Triton X-100, 0.1% Citrate buffer pH 6.0 [Dako]) for 30 min. Sections were then blocked with 10% serum (same species as the secondary antibodies) in staining buffer (PBS, 2.5 % BSA, 2 mM EDTA, 0.01 % NaN_3) for 1 hour at room temperature. Sections were incubated with primary antibodies (2.5-5 µg/ml) overnight at 4°C. Primary antibodies were extensively washed with PBS, and sections were incubated with secondary antibodies (2.5 µg/ml) diluted in staining buffer supplemented with 2.5% serum (same species as the secondary antibodies) for 4-6 hours at room temperature. Sections were extensively washed with PBS, nuclei were stained with Hoechst 33342 [Invitrogen], extensively washed and mounted using CC/Mount™ tissue mounting medium [Sigma]. We used the following primary antibodies: polyclonal rabbit anti-human/mouse CD3 [Dako]; polyclonal rabbit anti-MPO [Thermo Fisher Scientific]; and monoclonal rat anti-mouse Ly6G [clone: RB6-8C5, Thermo Fisher Scientific]. Primary antibodies were revealed using donkey anti-rabbit Alexa Fluor® 555 [Thermo Fisher Scientific]; goat anti-rat Alexa Fluor® 488
[Thermo Fisher Scientific]; or goat anti-rabbit Alexa Fluor® 647 [Thermo Fisher Scientific].For collagen analysis, air dried sections were washed in PBS and incubated in

Sirius red solution (i.e., 0.5 g of Direct Red 80 [Sigma] were diluted in 500 ml of saturated aqueous solution of picric acid) for 1 hour. Sections were extensively washed in PBS, dehydrated and mounted in DPX mounting medium [Sigma]. Sections were imaged under polarized light (birefringence of collagen fibres) and brightfield (area of the section). Aortic diameter was estimated using haematoxylin-eosin sections (average of 3 measurements per mouse) after measurement of the perimeter of the medial layer. Brightfield and polarized imaging were done using a DM6000B microscope [Leica] and analysed with accompanying software. Images analysis was performed using Adobe Photoshop CS5 and ImageJ [NIH]. Macroscopic imaging was realised using a M80 stereomicroscope [Leica], DS Camera Head DS-Fi1 [Nikon] and Digital Sight DS-L2 imaging controller [Nikon].

Supplemental Data 1: List of RESCAN collaborators

Professor PE Norman (University of Western Australia, Crawley, WA, Australia)

Mr S Parvin (Royal Bournemouth Hospital, Bournemouth, UK)

Mrs Hilary Ashton (St Richard's Hospital, Chichester, UK)

Mr RTA Chalmers (Edinburgh Royal Infirmary, Edinburgh, UK)

Mr JJ Earnshaw (Gloucestershire Royal Infirmary, Gloucester, UK)

Mr ABM Wilmink (Heart of England NHS Trust, Birmingham, UK)

Professor DJA Scott (University of Leeds, Leeds, UK)

Professor CN McCollum (University Hospital of South Manchester, Manchester, UK) Dr S Solberg (Rikshospitalet, Oslo, Norway)

Dr K Ouriel (Syntactx, New York, NY, USA)

Professor A Laupacis (St Michael's Hospital, Toronto, ON, Canada)

Dr M Vega de Céniga (Hospital de Galdakao, Bilbao, Spain)

Mr R Holdsworth (Stirling Royal Infirmary, Stirling, UK)

Dr L Karlsson (Gävle Hospital, Gävle, Sweden)

Professor JS Lindholt (Viborg Hospital, Viborg, Denmark)

Supplemental Data 2: List of Aneurysm Consortium collaborators

Benjamin J Wright (University of Leicester, Leicester, UK)

Suzannah Bumpstead (Wellcome Trust Sanger Institute, Cambridge, UK)

Stephen A Badger (Queens University, Belfast, UK)

Rachel E Clough (King's College London, London, UK)

Gillian Cockerill (St George's University of London, London, UK)

Hany Hafez (St Richard's Hospital, Chichester, UK)

D Julian A Scott (University of Leeds, Leeds, UK)

Marc A Bailey (University of Leeds, Leeds, UK)

Alberto Smith (King's College London, London, UK)

Matthew M Thompson (St George's University of London, London, UK) Jutta Palmen (University College London, London, UK)

Andrew J Smith (University College London, London, UK)

Jes S Lindholt (Viborg Hospital, Viborg, Denmark)

Declan T Bradley (Queens University, Belfast, UK)

Sarah Edkins (Wellcome Trust Sanger Institute, Cambridge, UK)

Rhian Gwilliam (Wellcome Trust Sanger Institute, Cambridge, UK) Sarah E Hunt (Wellcome Trust Sanger Institute, Cambridge, UK)

Simon Potter (Wellcome Trust Sanger Institute, Cambridge, UK)

Kathy Stirrups (Wellcome Trust Sanger Institute, Cambridge, UK) Jonathan Golledge (James Cook University, Townsville, Australia)

Paul E Norman (University of Western Australia, Crawley, Australia) Steve E Humphries (University College London, London, UK)

Panos Deloukas (Queen Mary University London, London, UK)

Supplemental Data 3: Power and sample size calculations

Power calculation for current study. Assumptions: Single nucleotide polymorphism effect – variance explained = 0.5% - 2% (corresponding to a per-allele effect size of 0.21mm/y – 0.43mm/year)), minor allele frequency=0.4, alpha=0.01 (two-sided) and power=80% or 90%. Calculated using Quanto. The variance explained by the genetic variant on AAA was estimated from a previous meta-analysis of genome-wide association studies of carotid intima media thickness and plaque (Bis et al. Nature Genetics 2011; 43: 940–947). SNPs from this meta-analysis explained ~1.1% of the trait variance. We used this as the basis of our power calculations and varied our assumptions that the variance explained ranged from 0.5-2%.

Variance explained (%) by genotype	Corresponding per allele effect size (mm/y)	Sample size required for	
		80% Power	90% Power
**0.5**	0.21	2330	2968
**0.7**	0.25	1711	2180
**1**	0.30	1162	1480
**2**	0.43	578	737

Sample size calculation estimating number of participants needed to be powered to see an effect size the same as that observed in the current study. **Sample size needed= 24,444**. Assumptions: minor allele frequency=0.4, population mean=1.8mm AAA growth/year (SD=2.3), effect size=0.06, and power=80%. Calculated using Quanto.

Supplementary Table 1: Summary of baseline characteristics by *IL6R* genotype

	Number copies of minor allele			
	0<br>n (%)	1<br>n (%)	2<br>n (%)	Total<br>n (%)
**Sex**				
Male	993 (38)	1,245 (48)	354 (14)	2,592 (100)
Female	109 (40)	130 (48)	31 (11)	270 (100)
**Age category**				
50-<70	436 (38)	543 (48)	158 (14)	1,137 (100)
≥70	664 (39)	832 (48)	227 (13)	1,723 (100)
**Current smoker**				
No	632 (39)	761 (47)	215 (13)	1,608 (100)
Yes	259 (38)	338 (49)	92 (13)	689 (100)
**Diabetes**				
No	939 (39)	1,167 (48)	325 (13)	2,431 (100)
Yes	128 (38)	156 (47)	50 (15)	334 (100)

Note: genotype not statistically associated with any baseline covariate (χ^2 p-value>0.05)

Supplementary Figure 1: Sex- and age-adjusted rate of change in AAA diameter

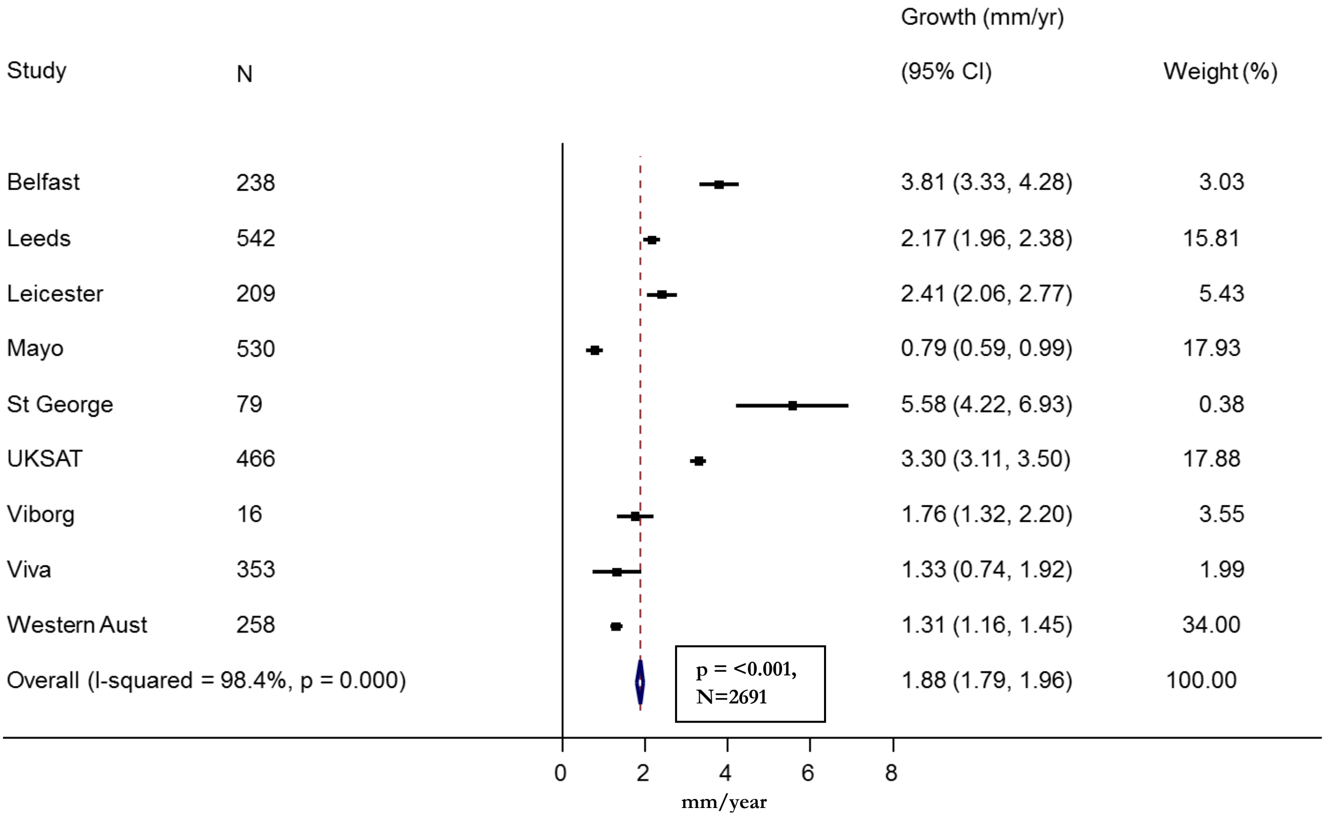

Supplementary Figure 2: Sex- and age-adjusted difference in baseline AAA size per copy of the *IL6R*-rs2228145 minor allele

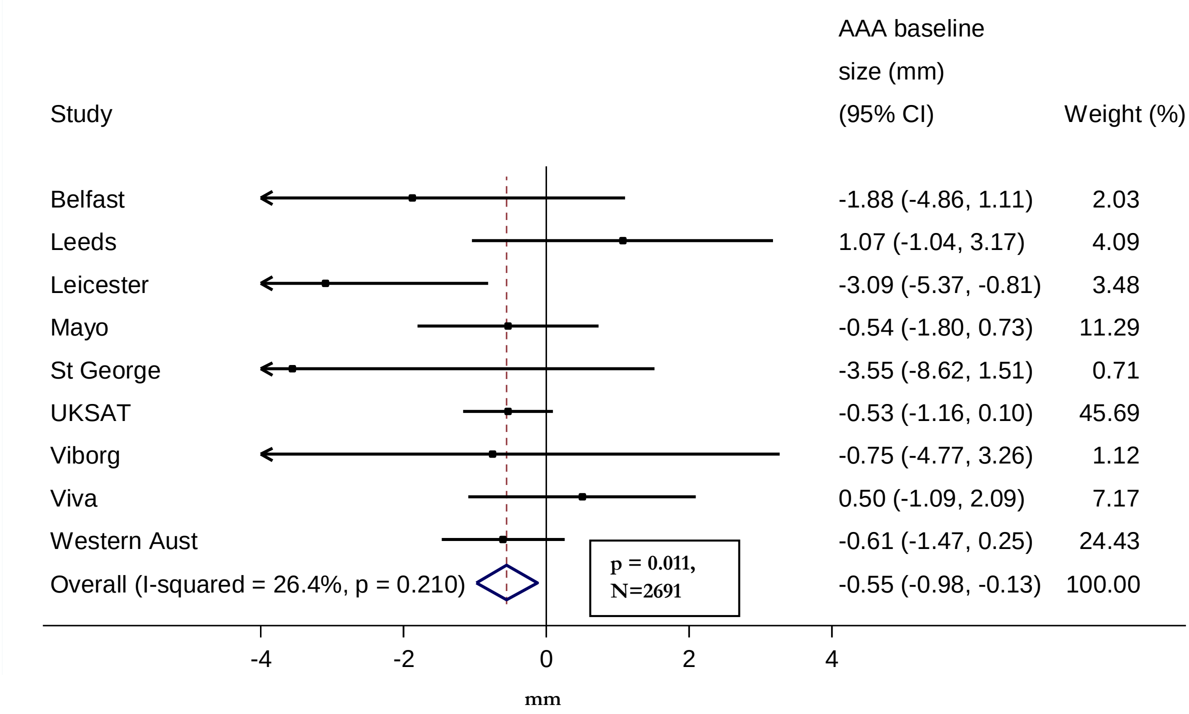

Supplementary Figure 3: Change in AAA growth rate (mm/year) per copy of the *IL6R*-rs2228145 minor allele, after adjustment for age, sex, current smoking, diabetes status, body mass index and measurement method

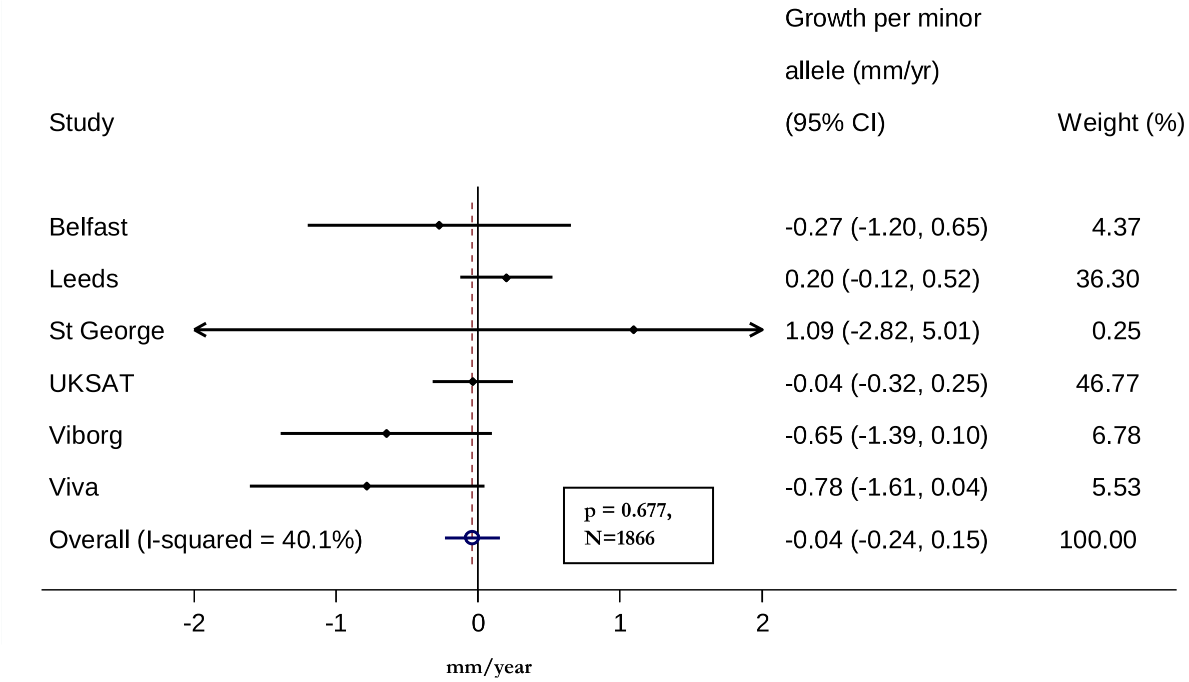

Notes: Missing values included in analysis as separate category. Only data from studies recording information across all covariates are included.

Supplementary Figure 4: Sex- and age-adjusted change in AAA growth rate (mm/year) per copy of the *IL6R*-rs2228145 minor allele, among people with small baseline size AAA

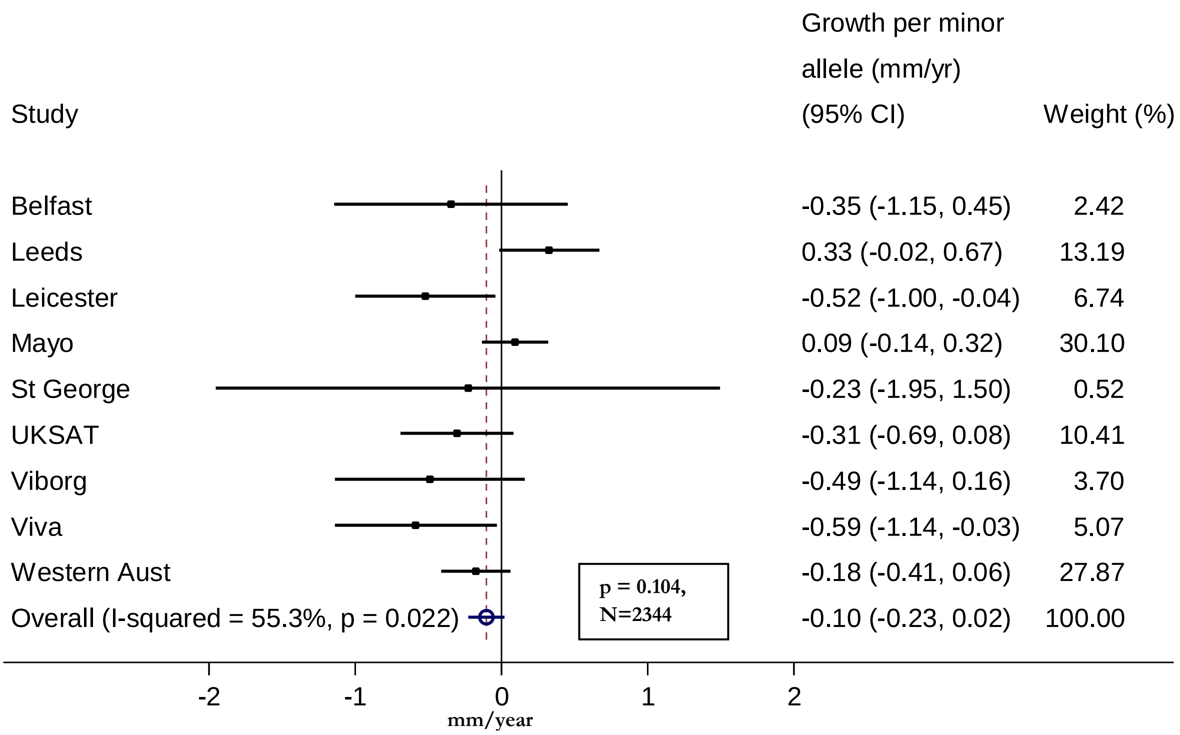

Notes: Small AAA defined as those with a baseline size of 30-44 mm. Aneurysms that grew larger than 44mm were censored after the first measurement >44mm.

Supplementary Figure 5: Sex- and age-adjusted change in AAA growth rate (mm/year) per copy of the *IL6R*-rs2228145 minor allele, among people with medium baseline size AAA

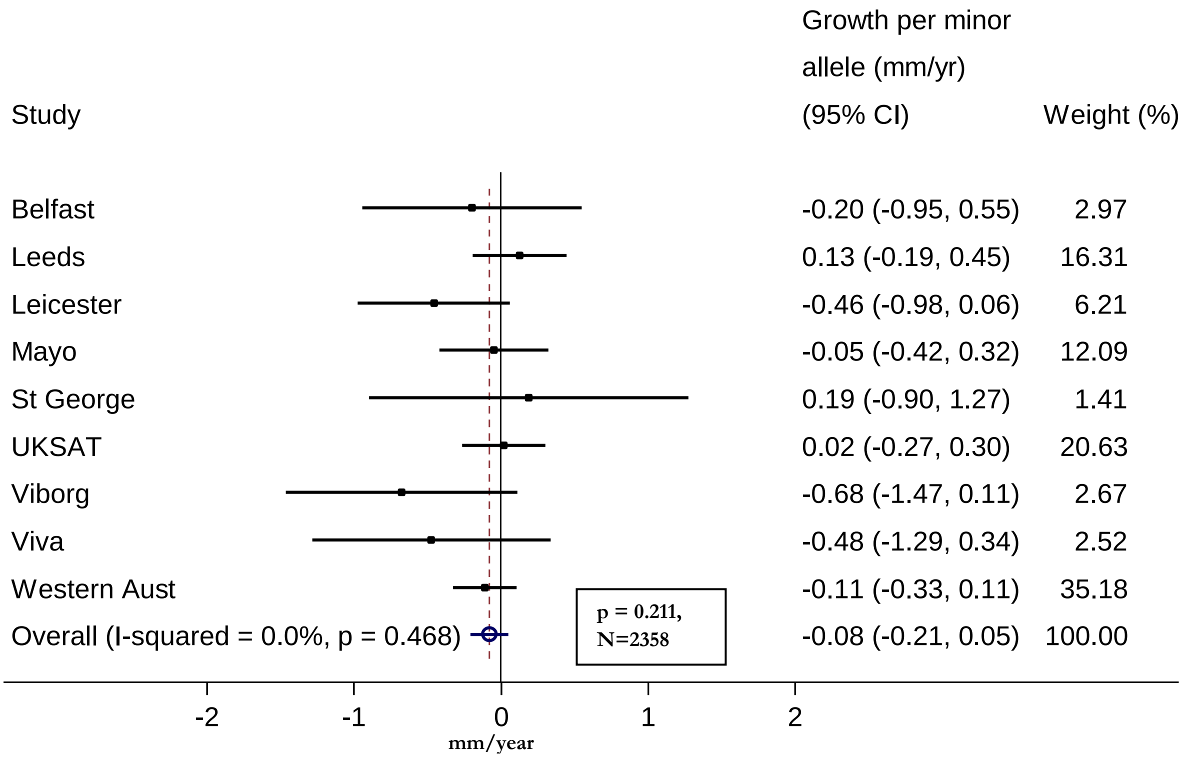

Notes: Medium AAA defined as those with a baseline size of 45-54 mm. Aneurysms that grew larger than 55mm were censored after the first measurement ≥55mm.

Supplementary Figure 6a: Sex- and age-adjusted hazards ratios (HR) for time to surgery threshold (AAA size≥55mm) per copy of the *IL6R*-rs2228145 minor allele

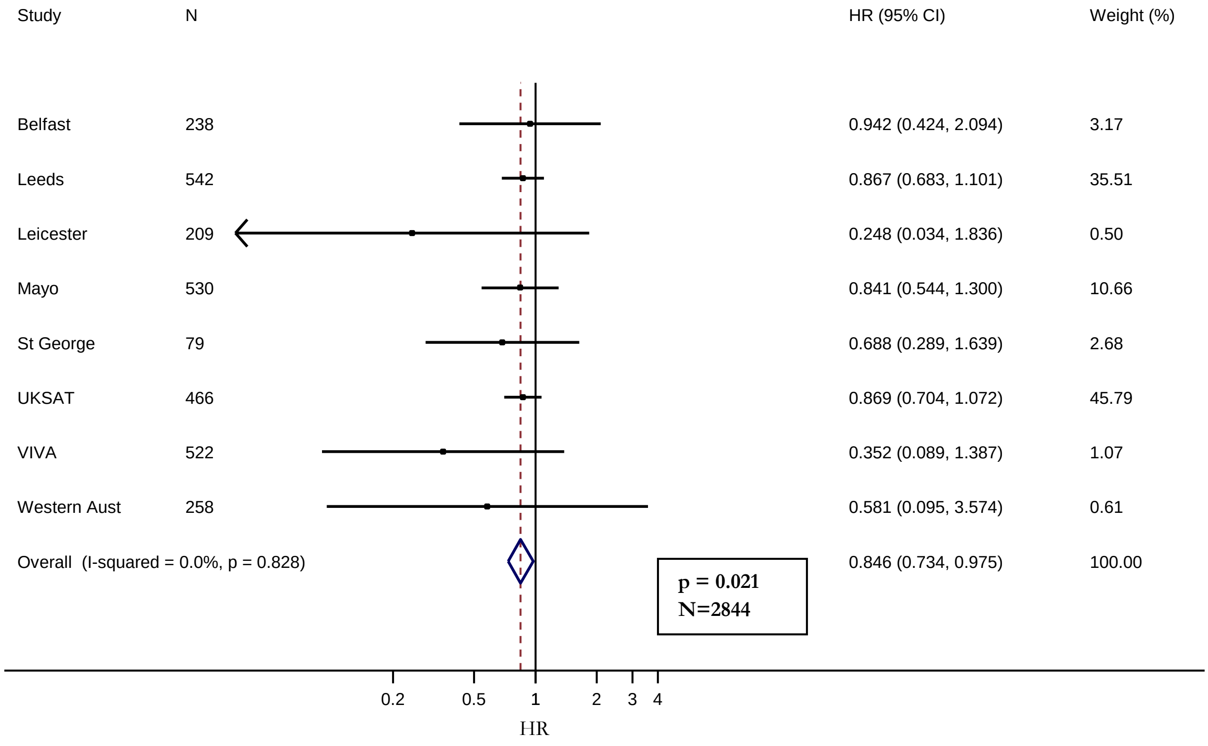

Notes: Viborg study excluded as only one participant reached the surgery threshold

Supplementary Figure 6b: Hazards ratios (HR) for time to surgery threshold (AAA size≥55mm) per copy of the *IL6R*-rs2228145 minor allele, adjusted for age, sex, current smoking, diabetes status, body mass index and measurement method

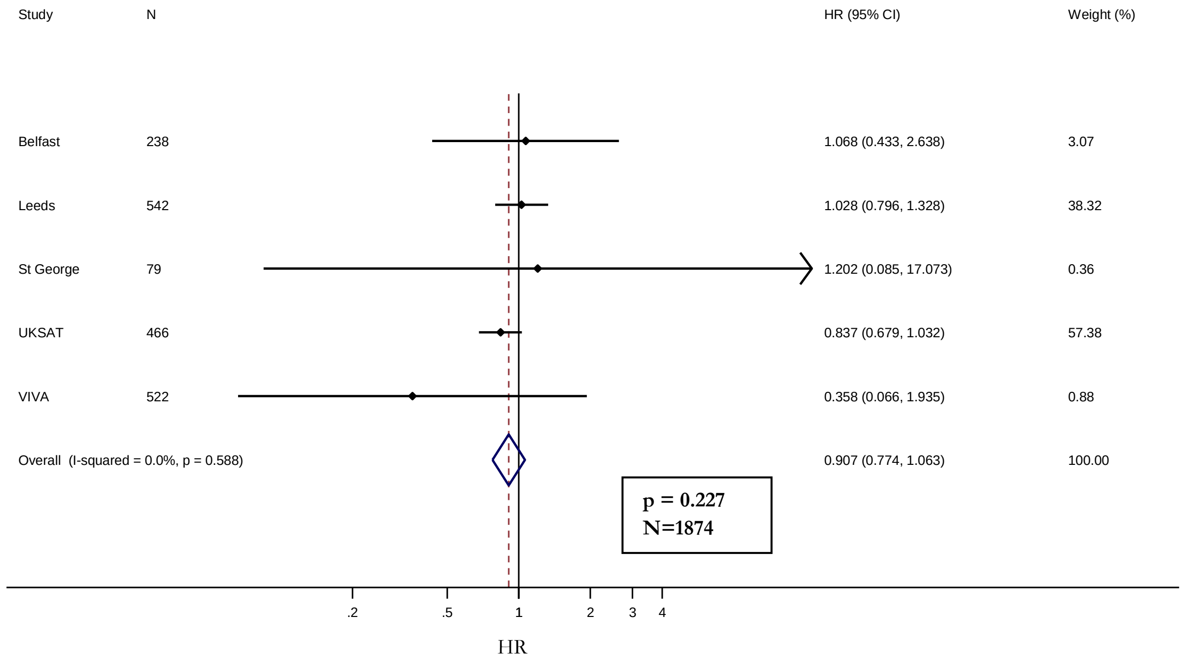

Notes: Missing values included in analysis as separate category. Only data from studies recording information across all covariates are included.

Supplementary Figure 7: Sex- and age-adjusted change in abdominal aortic aneurysm growth rate (mm/year) per copy of the *IL6R*-rs2228145 minor allele, including those single AAA measures

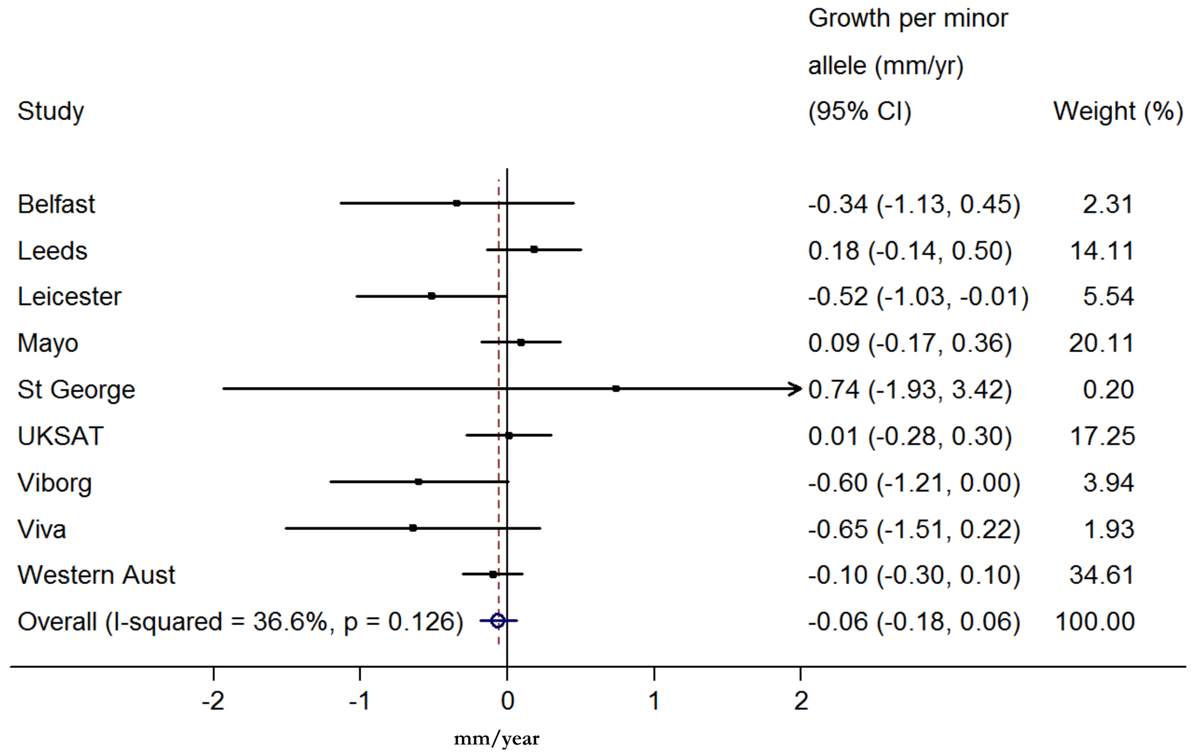

Supplementary Figure 8: Associations between the IL6R-rs2228145 variant and intermediate traits and cytokine profiles

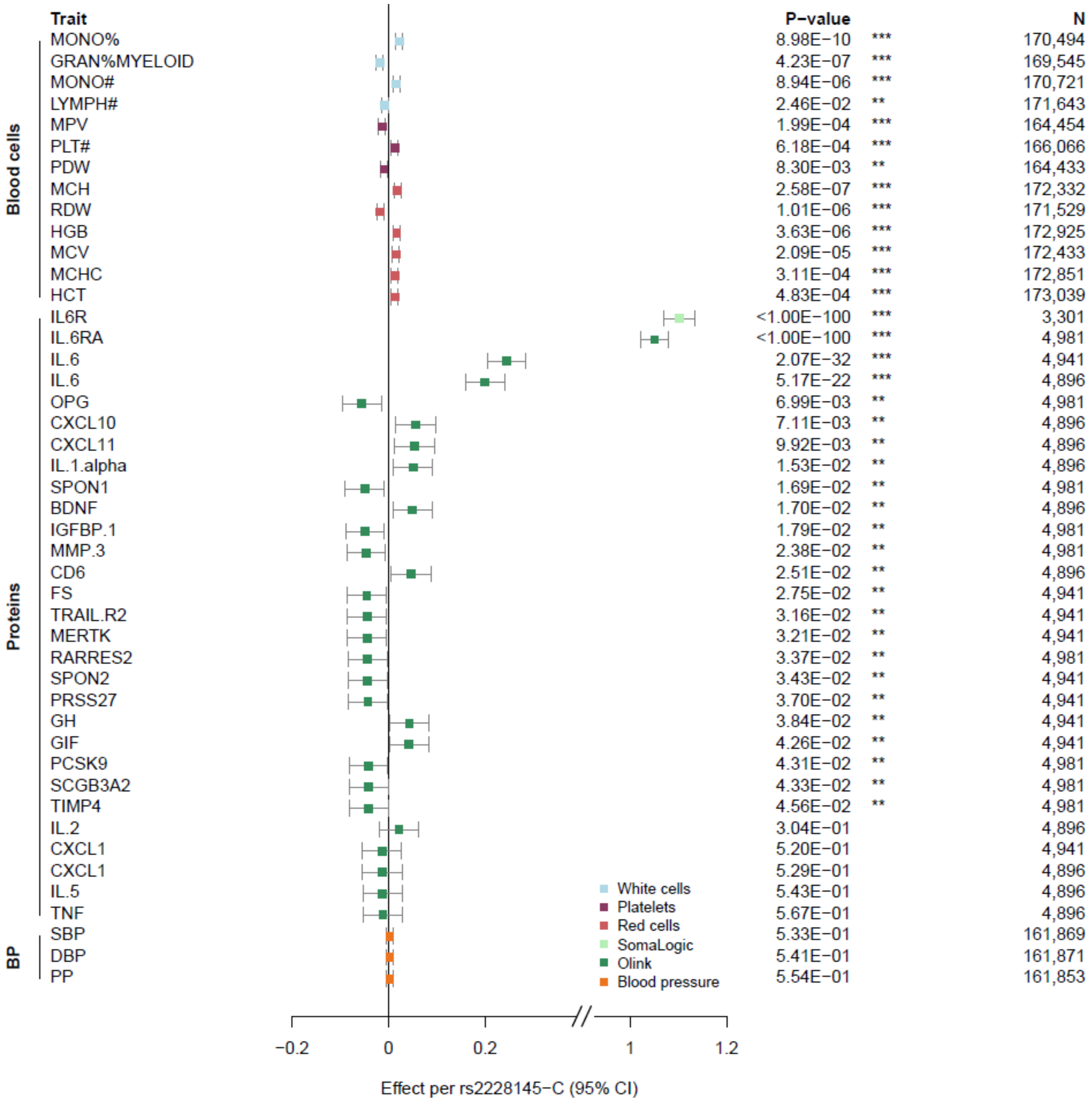

Notes: ***Bonferroni-corrected significance threshold (i.e. P<0.05/number of traits). **Nominal significance threshold (P<0.05). Error bars indicate standard error of effect size. Traits that did not reach significance were included to serve as a reference to the observations in the experimental mouse models. Proteins were measured using the assays provided by either SomaLogic (2,994 proteins) or Olink Bioscience (three 92-protein panels, i.e. 'inflammatory', 'cvd2' and 'cvd3'). A subset of ten proteins (including CXCL1 and IL-6) were assayed on more than one panel.

Abbreviations: **Blood cells:** (BASO+NEUT)#: Sum basophil neutrophil counts; (EO+BASO)#: Sum eosinophil basophil counts; (NEUT+EO)#: Sum neutrophil eosinophil counts; BASO#: Basophil count; BASO%: Basophil percentage of white cells; BASO%GRAN: Basophil percentage of granulocytes; EO#: Eosinophil count; EO%:

Eosinophil percentage of white cells; EO%GRAN: Eosinophil percentage of granulocytes; GRAN#: Granulocyte count; GRAN%MYELOID: Granulocyte percentage of myeloid white cells; HCT: Hematocrit; HGB: Hemoglobin concentration; HLSR#: High light scatter reticulocyte count; HLSR %: High light scatter reticulocyte percentage of red cells; IRF: Immature fraction of reticulocytes; LYMPH#: Lymphocyte count; LYMPH%: Lymphocyte percentage of white cells; MCH: Mean corpuscular haemoglobin; MCHC: Mean corpuscular hemoglobin concentration; MCV: Mean corpuscular volume; MONO#: Monocyte count; MONO%: Monocyte percentage of white cells; MPV: Mean platelet volume; MYELOID#: Myeloid white cell count; NEUT#: Neutrophil count; NEUT%: Neutrophil percentage of white cells; NEUT%GRAN: Neutrophil percentage of granulocytes; PCT: Plateletcrit; PDW: Platelet distribution width; PLT#: Platelet count; RBC#: Red blood cell count; RDW: Red cell distribution width; RET#: Reticulocyte count; RET%: Reticulocyte fraction of red cells; WBC#: White blood cell count. **Blood pressure (BP):** DBP: Diastolic blood pressure; PP: Pulse pressure; SBP: Systolic blood pressure.

References:

1. Sakalihasan N, et al. Abdominal aortic aneurysm. *Lancet.* 2005;365:1577-1589.
2. Higgins JP, Thompson SG. Quantifying heterogeneity in a meta-analysis. *Stat Med.* 2002;21:1539-1558.
3. Aggarwal S, et al. Abdominal aortic aneurysm: A comprehensive review. *Experimental & Clinical Cardiology.* 2011;16:11-15.
4. Astle WJ, et al. The Allelic Landscape of Human Blood Cell Trait Variation and Links to Common Complex Disease. *Cell.* 2016;167:1415-1429.e1419.
5. Surendran P, et al. Trans-ancestry meta-analyses identify rare and common variants associated with blood pressure and hypertension. *Nat Genet.* 2016;48:1151-1161.
6. Sun BB, et al. Genomic atlas of the human plasma proteome. *Nature.* 2018;558:73-79.
7. Wang Y, et al. TGF-beta activity protects against inflammatory aortic aneurysm progression and complications in angiotensin II-infused mice. *J Clin Invest.* 2010;120:422-432.
8. Lareyre F, et al. TGFβ (Transforming Growth Factor-β) Blockade Induces a Human-Like Disease in a Nondissecting Mouse Model of Abdominal Aortic Aneurysm. *Arteriosclerosis, Thrombosis, and Vascular Biology.* 2017.
9. Okazaki M, et al. Characterization of anti-mouse interleukin-6 receptor antibody. *Immunol Lett.* 2002;84:231-240.
10. Katsume A, et al. Anti-interleukin 6 (IL-6) receptor antibody suppresses Castleman's disease like symptoms emerged in IL-6 transgenic mice. *Cytokine.* 2002;20:304-311.
11. Jostock T, et al. Soluble gp130 is the natural inhibitor of soluble interleukin-6 receptor transsignaling responses. *Eur J Biochem.* 2001;268:160-167.
