# Supplementary material for: Interleukin-6 Receptor Signaling and Abdominal Aortic Aneurysm Growth Rates
Source: Circ Genom Precis Med. 2019 Feb 19;12(2):e002413. doi: 10.1161/CIRCGEN.118.002413 (PMC6383754; doi:10.1161/CIRCGEN.118.002413)
Supplement: Supplementary file 2 [file hcg-12-e002413-s002.pdf]

Doi: 10.1161/CIRCGEN.118.002413

Interleukin-6 Receptor Signalling and Abdominal Aortic Aneurysm Growth Rates

Running title: *Paige & Clément et al.; IL-6R Signalling and AAA Growth Rates*

Ellie Paige, PhD[1,5]*; Marc Clément, PhD[2]*; Fabien Lareyre, MD, PhD[2-4]; Michael Sweeting, PhD[5,6]; Juliette Raffort, MD, PhD[2-4]; Céline Grenier, PhD[2]; Alison Finigan[2]; James Harrison, BA[2]; James E. Peters, MBChB, PhD[5,7]; Benjamin B. Sun, PhD[5]; Adam S. Butterworth, PhD[5,7,8]; Seamus C. Harrison, PhD, FRCS[7,9]; Matthew J. Bown, MBBCh, PhD, FRCS[9]; Jes S. Lindholt, MD, PhD, DMSci[10]; Stephen A. Badger, MD, MCh, FRCSEd[11]; Iftikhar J. Kullo, MD[12]; Janet Powell, MD, PhD[13]; Paul E. Norman, DS[14]; D. Julian A. Scott, MD, MBChB, FRCS, FRCSEd, FEBVS[15,16]; Marc A. Bailey, MBChB, PhD[15,16]; Stefan Rose-John, PhD[17]; John Danesh, MBChB, DPhil, FRCP, FFPH, FMedSci[5,7,8,18]; Daniel F. Freitag, PhD[5,7]†; Dirk S. Paul, PhD[5,7]†; Ziad Mallat, MD, PhD[2,7,19]†

[1]National Ctr for Epidemiology & Population Health, Research School of Population Health, The Australian National Univ, Canberra, Australia; [2]Division of Cardiovascular Medicine, [5]BHF Cardiovascular Epidemiology Unit, Dept of Public Health & Primary Care, Univ of Cambridge, Cambridge, UK; [3]Université Côte d'Azur, Institut National de la Sante et de la Recherche Medicale, Centre Mediterranéen de Recherche Moleculaire; [4]Univ Hospital of Nice, Nice, France; [6]Dept of Health Sciences, [9]Dept of Cardiovascular Sciences & NIHR Leicester Biomedical Research Ctr, Univ of Leicester, Leicester; [7]British Heart Foundation Centre of Excellence, Univ of Cambridge School of Clinical Medicine; [8]NIHR Blood & Transplant Research Unit in Donor Health and Genomics, Cambridge, UK; [10]Dept of Cardiovascular & Thoracic surgery, Elitary Reserach Ctr of Individualised Medicine in Arterial Disease, Odense Univ Hospital, Odense, Denmark; [11]Regional Vascular Surgery Unit, Belfast Health & Social Care Trust, Belfast, UK; [12]Dept of Cardiovascular Medicine & Gonda Vascular Ctr, Mayo Clinic, Rochester, MN; [13]Faculty of Medicine, Dept of Surgery & Cancer, Imperial College London, London, UK; [14]Medical School, Univ of Western Australia, Perth, Australia; [15]Leeds Vascular Institute, Leeds General Infirmary; [16]Leeds Institute of Cardiovascular & Metabolic Medicine, School of Medicine, Univ of Leeds, Leeds, UK; [17]Dept of Biochemistry, Christian-Albrechts-Univ, Kiel, Germany; [18]Dept of Human Genetics, Wellcome Sanger Institute, Hinxton, UK; [19]Institut National de la Santé et de la Recherche Médicale, Paris Cardiovascular Research Center, France
*Joint first authors / †Joint senior authors

Correspondence:

Professor Ziad Mallat

Department of Medicine,

University of Cambridge

Cambridge, CB2 0QQ

United Kingdom

Tel: +44 (0) 1223 768678

E-mail: zm255@medschl.cam.ac.uk

Journal Subject Terms: Animal Models of Human Disease; Inflammation; Epidemiology; Genetic, Association Studies; Aneurysm.

Doi: 10.1161/CIRCGEN.118.002413

Abstract:

Background: The Asp358Ala variant (rs2228145; A>C) in the interleukin-6 receptor (*IL6R*) gene has been implicated in the development of abdominal aortic aneurysms (AAAs), but its effect on AAA growth over time is not known. We aimed to investigate the clinical association between the *IL6R*-Asp358Ala variant and AAA growth, and to assess the effect of blocking the IL-6 signalling pathway in mouse models of aortic aneurysm rupture or dissection.

Method: Using data from 2,863 participants with AAA from nine prospective cohorts, age- and sex-adjusted mixed-effects linear regression models were used to estimate the association between the *IL6R*-Asp358Ala variant and annual change in AAA diameter (mm/year). In a series of complementary randomised trials in mice, the effect of blocking the IL-6 signalling pathways was assessed on plasma biomarkers, systolic blood pressure, aneurysm diameter and time to aortic rupture and death.

Results: After adjusting for age and sex, baseline aneurysm size was 0.55mm (95% confidence interval [CI]: 0.13, 0.98mm) smaller per copy of the minor allele [C] of the Asp358Ala variant. Change in AAA growth was -0.06mm per year [-0.18, 0.06] per copy of the minor allele; a result that was not statistically significant. Although all available worldwide data were used, the genetic analyses were not powered for an effect size as small as that observed. In two mouse models of AAA, selective blockage of the IL-6 trans-signalling pathway, but not combined blockage of both, the classical and trans-signalling pathways, was associated with improved survival ($p<0.05$).

Conclusions: Our proof-of-principle data are compatible with the concept that IL-6 trans-signalling is relevant to AAA growth, encouraging larger-scale evaluation of this hypothesis.

American Heart Association
Circulation: Genomic and Precision Medicine

Key words: interleukin-6 receptor; abdominal aortic aneurysm; inflammation; genetics

Introduction

Abdominal aortic aneurysms (AAAs) are defined as an enlargement of the aorta to ≥30mm diameter. They usually grow asymptomatically until rupture occurs, after which the survival of affected individuals is less than 20%.[1] AAAs typically occur in mid-to-later life and more commonly in men (prevalence of 1-2%[2, 3] compared to <1% in women[4]). Current standard of care is surgical intervention, either open surgery or endovascular repair. However, due to surgical risks vs. benefits, such interventions are generally recommended only for people with larger AAAs (diameter ≥55mm or >40mm and enlarging >10mm/year).[5, 6] The growth rates of AAAs vary considerably between individuals[7] and there is currently a lack of therapeutic options to slow or halt progression of AAAs.[8] Inflammatory processes in the vessel wall may contribute to the progression of AAAs.[9-11] For example, levels of circulating inflammatory markers including interleukin (IL)-6 are higher in prevalent AAA cases than controls[12] and correlate with the size of AAA in cross-sectional studies.[13]

American Heart Association. Circulation: Genomic and Precision Medicine

IL-6 is a central coordinator of inflammatory responses by controlling the systemic inflammatory response in the liver and the activation and differentiation of leukocyte subsets, including macrophages and T cells. IL-6 signalling occurs in two different modes, termed “classical” (cis-) and trans-signalling. In classical signalling, binding of IL-6 to the membrane-bound IL-6 receptor (mIL-6R) induces homodimerization with its co-receptor gp130, resulting in the phosphorylation of the transcription factors STAT3 and STAT1.[14, 15] Hence, classical signalling is dependent on the membrane-bound form of the IL-6 receptor and occurs only in leukocyte subsets and hepatocytes that express this molecule. By contrast, trans-signalling occurs through a circulating soluble form of IL-6R (sIL-6R), which, if bound to IL-6, is able to stimulate cells expressing gp130, even in the absence of mIL-6R.[14, 16] Because gp130 is almost

ubiquitously expressed, IL-6 trans-signalling can occur in virtually any cell, although is probably active only during conditions of immunological stress.[14, 16]

A non-synonymous variant (Asp358Ala; rs2228145 A>C) in the *IL6R* gene, encoding the IL-6 receptor, plays a critical role in IL-6 signalling. The minor allele [C] of this variant is associated with a reduced risk of several chronic conditions including coronary heart disease,[17] atrial fibrillation,[18] rheumatoid arthritis,[19] type 1 diabetes,[20] but an increased risk of asthma.[21] The variant results in more efficient proteolytic cleavage of mIL-6R, thereby reducing levels of mIL-6R and dampening classical signalling.[22, 23] Conversely, the variant increases levels of sIL-6R, although the exact effects of the variant on the trans-signalling pathway are unknown.[20] A Mendelian randomisation study has previously implicated the rs2228145 in the causal pathway of AAA, with the minor allele [C] showing a protective effect for the risk of AAA and a combined endpoint of rupture or surgical intervention.[12] Licenced drugs are available that target the IL-6/IL-6 receptor pathway. However, evidence is needed that this pathway is associated with aneurysm progression or rupture in order to encourage repurposing drugs for use in patients with known AAA.

American Heart Association.
Circulation: Genomic and Precision Medicine

The aims of this study were to: (1) to assess and quantify the effect of the functional *IL6R* variant on the progression of AAAs in population cohorts with prospective follow-up and standardized repeated measurements of AAA diameter; and (2) estimate the effect of blocking the IL-6 signalling pathway (i.e., either both classical and trans-signalling pathways or specifically the trans-signalling pathway) on time to aneurysm rupture in mouse models.

Methods

Details on the materials and methods are available in the Supplemental Material. Because of the sensitive nature of the data collected for this study, requests to access the human AAA datasets from qualified researchers trained in human subject confidentiality protocols may be sent to the study leaders of each cohort. The experimental data that support the findings of this study are available from the corresponding author upon reasonable request. Each human AAA cohort was approved by a research ethics committee and all participants gave informed consent. Animal experiments were approved by the UK Home Office and performed under PPL PA4BDF775. The care and use of all mice in this study was carried out in accordance with the UK Home Office regulations under the Animals (Scientific Procedures) Act 1986.

American Heart Association. Circulation: Genomic and Precision Medicine

Results

Association of IL6R-Asp358Ala with AAA growth rate

We studied a total of 2,863 participants across nine prospective cohorts, 91% of whom were men (mean age of 72 years; Table 1). AAA size was on average 43mm at baseline and participants had an average of four scans across an average of three years (Table 1). A summary of baseline characteristics by *IL6R*-rs2228145 (or proxy) genotype is given in Supplementary Table 1. On average, aneurysms grew by 1.88mm per year (95% CI: 1.79, 1.96; Supplementary Figure 1). Baseline aneurysm size was 0.55mm (0.13, 0.98) smaller per copy of the minor allele [C] (Supplementary Figure 2). Among those with at least two measurements of aneurysm size (n=2,154), there was no statistically significant growth rate in AAA per minor allele (growth per minor allele= -0.06mm/year [-0.18, 0.06]) (Figure 1). All association analyses were adjusted for

age and sex. Results were similar after adjustment for current smoking status, diabetes status, body mass index and aneurysm measurement method (Supplementary Figure 3).

Similar results were observed when the analysis was restricted to those with a small aneurysm at baseline (growth= -0.10 mm/year [-0.23, 0.02] per copy of the minor allele; Supplementary Figure 4) or medium aneurysm at baseline (growth= -0.08 mm/year [-0.21, 0.05] per copy of the minor allele; Supplementary Figure 5). We did observe an association between the Asp358Ala variant and time to surgery threshold after adjusting for age and sex (hazards ratio (HR)= 0.85 [0.73, 0.98] per copy of the minor allele; Supplementary Figure 6a). The HR was in the same direction but became statistically non-significant in the subset of studies were ving and surviving an event, may have biased the results towards the null. ADDIN EN.CITE ADDI NEN.CITE.DATA⊥ !! igure6b).Overallchangein AAA growth remained the same when individuals with only a single measure of aneurysm size were included in the model (n=2,691, growth=-0.06 mm/year [-0.18, 0.06]; Supplementary Figure 7) [Table 1]

Inhibition of IL-6 signalling pathway in angiotensin II + anti-TGFβ mouse model

We next tested the effect of blocking the IL-6 pathway in two distinct, previously characterised mouse models of AAA (Methods). In the angiotensin II + anti-TGFβ model, mice infused with anti-IL-6R (blocking both classical and trans-signalling pathways) demonstrated a significant increase of plasma concentration of IL-6, as compared to isotype treated mice, and this difference was sustained over the course of the experiment (Figure 2A). We observed a reduction in plasma concentrations of serum amyloid A (SAA), a protein expressed in response to inflammation, after blocking IL-6R compared to the control mice (Figure 2B). Blocking IL-6R significantly reduced plasma concentration of IL-2 before and after the infusion and reduced

American Heart Association
Circulation: Genomic and Precision Medicine

concentration of IL-5 and chemokine ligand 1 (CXCL1) after the infusion (Figures 2A). After anti-IL-6R treatment, systolic blood pressure was significantly lower after the infusion compared to control treatment (Figure 2C). However, there was no significant difference in rate of aneurysm rupture between the anti-IL-6R treated and control groups (Figure 2D). Since there was no observed association with AAA rupture, we did not further assess the effect of blocking the IL-6R pathway on AAA growth.

Selectively blocking the IL-6 trans-signalling pathway using sgp130Fc did not change the concentration of IL-6 (Figure 3A) or SAA (Figure 3B), but significantly induced IL-5 and reduced TNFα plasma concentration (Figure 3A). Although we observed no difference in systolic blood pressure between mice treated with sgp130Fc and the control mice (Figure 3C), there was a significant reduction in aneurysm rupture after sgp130Fc treatment compared to control treatment (Figure 3D).

American Heart Association. Circulation: Genomic and Precision Medicine

Inhibition of IL-6 signalling pathway in elastase + anti-TGFβ mouse model

Using the elastase + anti-TGFβ model, we found that blockage of the IL-6R pathway using anti-IL-6R resulted in significantly increased mortality (Figure 4A) induced by aortic rupture (Figure 4B) but there was no change in the diameter of the aneurysm at the end of the experiment (Figure 4C). αSMA^+ density in the media was similar in the two groups of mice (data not shown). There was also no change in the collagen content of the aortic wall (Figure 4D) or the recruitment of myeloperoxidase positive (MPO^+) cells (Figure 4E), but treatment with anti-IL-6R significantly enhanced the recruitment of $CD3^+$ T cells in the aortic wall (Figure 4F).

Blocking only the IL-6 trans-signalling pathway using sgp130Fc significantly increased survival (Figure 5A) by reducing aortic ruptures (Figure 5B), although at the end of the experiment there was no change in the aortic diameter between the treated and control mice

(Figure 5C). Histological analysis of aortic samples revealed a significant increase in the collagen content of the arterial wall (Figure 5D) but no differences in αSMA^{+} density (data not shown), Ly6G^{+} (Figure 5E) and CD3^{+} T cell accumulation (Figure 5F) after sgp130Fc infusion, as compared to the control mice. Table 2 summarises the results of the different mouse models with a comparison to the human genetic data.

Evaluation of the effect of IL6R-rs2228145 on a range of cardiovascular markers

As would be expected, the minor allele of *IL6R*-rs2228145 was associated with increased plasma concentrations of IL-6 and sIL-6R. The variant was also associated with increased monocyte count, after correcting for multiple comparisons ($p<1.389x10^{-3}$). At a nominal significance level ($p<0.05$), rs2228145-C was associated with reduced lymphocyte count, increased levels of the cytokines CXCL10, CXCL11 and IL1α, as well as CD6 (an important regulator of T cells), and reduced levels of MMP3 (a matrix metalloproteinase), TIMP4 (a metalloproteinase inhibitor), OPG (osteoprotegerin) and IGFBP1 (Supplementary Figure 8). In our analysis, the effects on plasma IL-2, IL-5, and CXCL1 levels, as well as on blood pressure, were not statistically significant.

[Table 2]

Discussion

In a combined analysis of the available worldwide clinical genetic data on AAA growth, we observed no statistically significant decrease in annual AAA growth rates for carriers of the minor allele of the Asp358Ala variant (rs2228145) in the *IL6R* gene. While we did observe a 15% decrease in the rate of reaching the surgery threshold of ≥55mm (HR=0.85 [0.73, 0.98] per copy of the minor allele), people with copies of the *IL6R*-Asp358Ala variant also had, on

American Heart Association
Circulation: Genomic and Precision Medicine

average, smaller baseline aneurysm diameters. Although we tried to account for this by allowing baseline hazards to vary depending on initial aneurysm size, some residual confounding is possible and could explain the observed results. In experimental data from mouse models, we found that selective blockade of the IL-6 trans-signalling pathway was associated with decreased aortic rupture and death. In exploratory analyses of cardiovascular and inflammatory biomarkers in healthy participants, we found that rs2228145-C was inversely associated with plasma levels of osteoprotegerin, matrix metalloproteinase-3 and metalloproteinase inhibitor-4 ($p<0.05$). Osteoprotegerin has previously been shown to promote matrix metalloprotease release from monocytes and vascular smooth muscle cells,[31, 32] and aberrant aortic extracellular matrix remodelling has been suggested to play a key role in the pathogenesis of AAA.[33] However, we note that further studies are needed to validate our biomarker data. Taken together, these human genetic, biomarker, and experimental murine findings are compatible with the concept that IL-6 trans-signalling is relevant to AAA growth, encouraging larger-scale evaluation of this hypothesis.

American Heart
Circulation: Genomic and Precision Medicine

If increased availability of sIL-6R results in a dampening of the IL-6 trans-signalling pathway, this may explain potential protective effects in AAA and is consistent with previously observed protective effects in mouse models of sepsis[34] and pancreatic and lung failure.[35] As we found a consistent pattern of results when the trans-signalling pathway was selectively blocked in the mouse models, it suggests that this pathway could have a detrimental effect on AAA growth (Figure 6). For example, the minor allele of the rs2228145 variant may result in a local reduction of IL-6 trans-signalling in the abdominal vasculature, reducing AAA risk[12] and, perhaps, AAA growth rates. Our observation of only a small, but statistically insignificant, decrease in annual AAA growth rates does not preclude meaningful clinical effects, since the

growth rate reduction estimated from natural genetic variation does not necessarily relate to the magnitude of the benefit that might result from pharmacological treatment directed at the IL-6 trans-signalling pathway.[36] Selective blocking of the IL-6 trans-signalling pathway using sgp130Fc is being investigated in phase II clinical trials in patients with inflammatory bowel disease.[14]

Nevertheless, apparent inconsistencies in our findings require further elucidation. For example, blockade of both the classic and trans-signalling IL-6 pathways using the animal-equivalent (MR16-1) of tocilizumab had no effect on AAA rupture in the angiotensin II + anti-TGFβ model, but it was associated with decreased survival in the elastase + anti-TGFβ model. These different outcomes could be explained by differences in the development of AAA in the different mouse models. The primary process of "aneurysm" formation in the angiotensin model is a medial dissection, which may be accentuated by elevations in blood pressure (even though high blood pressure is not the primary cause of medial dissection). Hence, the potential protection afforded by MR16-1 antibody in this model can at least in part be attributed to the significant reduction of blood pressure. In contrast, the elastase model does not involve medial dissection or elevations in blood pressure, but induces progressive remodelling, dilatation, and eventually transmural rupture of the artery wall, better mimicking AAA progression in humans.[37, 38]

American Heart Association
Circulation: Genomic and Precision Medicine

If selective blockade of the IL-6 trans-signalling pathway results in decreased aortic rupture, as suggested by our murine data, one might expect that blocking both the classic and trans-signalling pathways would also result in decreased aortic rupture. However, we did not observe such a finding, perhaps due to competing downstream actions of the classic and trans-signalling pathways. Such an explanation is consistent with our finding that IL-5 levels were

increased and TNFα levels decreased when trans-signalling was selectively blocked, whereas blockade of both classic and trans-signalling pathways led to reductions in IL-5 levels and no changes in TNFα. Our findings suggest that selective blockade of the IL-6 trans-signalling pathway, compared to blockade of both IL-6 signalling pathways, results in different downstream cytokine profiles and potentially different effects on AAA progression. It is also possible that the blocking of both the classical signaling cascade (considered to have protective and regenerative cellular effects) and trans-signaling cascade (considered to have pro-inflammatory effects) cancelled each other out, leading to no detectable effect on AAA rupture. Further studies are needed to replicate and further characterise our findings.

We undertook a range of sensitivity analyses to test assumptions underlying our longitudinal human genetic studies. We studied complementary murine models of AAA, including the elastase + anti-TGFβ mouse model that has been shown to more closely mimic the AAA growth and rupture patterns seen in humans. To generate new mechanistic hypotheses, we conducted exploratory studies of the *IL6R*-Asp358Ala variant in relation to cardiovascular and inflammatory plasma biomarkers recorded in healthy participants. The experimental mouse studies were conducted under severe conditions in which TGFβ was blocked. Although any effect of IL-6R signalling might have been easier to observe in a less severe model (aortic dilatation without rupture), a protective effect of the intervention in that setting would not provide assurance that the intervention will also be protective in a more severe model (aortic rupture). A treatment that limits aortic dilatation but does not reduce the risk of aortic rupture would have limited clinical relevance.

Our study had potential limitations. It was powered to detect reductions in aneurysm growth of ~0.21mm per year or larger, much greater than the observed non-significant decrease

American Heart Association
Circulation: Genomic and Precision Medicine

of 0.06mm per year. Future studies powered to see an effect the same size as that observed in the current study would need to recruit an additional ~21,500 participants (total participants needed=24,444, Supplemental Material). This is unlikely to be achievable in the near future; alternative study methods using a composite phenotype for disease progression may be needed. Index event and survival bias, in which participants are selected into the study based on both having and surviving an event, may have biased the results towards the null.[39] However, this bias is likely to be small (<10%).[39] Further, ultrasound, the primary method used to assess AAA diameter in the included studies, has a margin of error of 2-3mm,[40] greater than the annual rate of aneurysm growth, making changes in growth difficult to detect. This might be why we observed an association between the *IL6R*-Asp358Ala variant and time to surgery threshold of ≥55mm but not when looking at continuous change in AAA size. Although we examined rupture rather than aortic diameter as the outcome in the mouse experimental models, our published data indicate that aortas that rupture have larger diameters or faster diameter progression than the ones that do not rupture.[30]

American Heart Association
Circulation: Genomic and Precision Medicine

It is also uncertain how well the results of our animal models translate to clinical disease. For example, an important difference is that IL-6 blockage is initiated before or at the time of disease development in the mice models of AAA, thereby not truly mimicking the treatment effects expected in humans, in which drugs to block IL-6 pathway would be started after disease onset. Blockade of the IL-6 signalling pathways in the angiotensin II + anti-TGFβ mouse model resulted in reproducible reductions in systolic blood pressure. Although tocilizumab has been anecdotally reported to improve pulmonary hypertension in Castleman's disease,[41, 42] the rs2228145 variant was not associated with changes in systemic blood pressure in healthy participants in a genome-wide association study. A large-scale randomized trial found no

difference in the number of hypertension events reported in those using tocilizumab compared to placebo.[43, 44] Thus, the acute responses to pharmacological doses of angiotensin II in the mouse model may not faithfully reproduce the human setting of AAA.

Our study may have clinical implications. Tocilizumab is currently indicated in a few disease settings, including rheumatoid arthritis and giant cell arteritis, both of which are associated with an increased risk of aortic aneurysm.[45, 46] The development of coronary artery aneurysms has also been reported in a non-placebo-controlled pilot study of tocilizumab in children with Kawasaki's Disease.[47] Findings from this pilot study in children, combined with our finding that blocking the IL-6 pathway using the animal-equivalent of tocilizumab was associated with decreased survival in the elastase + anti-TGFβ model, suggests that patients treated with tocilizumab for conditions associated with aortic aneurysm development should possibly be monitored for AAA.

American Heart Association.
Circulation: Genomic and Precision Medicine

In conclusion, our proof-of-principle data are potentially compatible with the concept that IL-6 trans-signalling is relevant to AAA growth, encouraging larger-scale evaluation of this hypothesis.

Acknowledgments: We thank Chugai Pharmaceutical Co., Ltd. for providing the MR16-1. We thank Tao Jiang and Praveen Surendran (Cardiovascular Epidemiology Unit, University of Cambridge) for providing access to genomic datasets.

Sources of Funding: The Cardiovascular Epidemiology Unit is supported by the UK Medical Research Council (MR/L003120/1), British Heart Foundation (RG/13/13/30194), and National Institute for Health Research [Cambridge Biomedical Research Centre at the Cambridge University Hospitals NHS Foundation Trust]. The views expressed are those of the authors and not necessarily those of the NHS, the NIHR or the Department of Health and Social Care.. SRJ was supported by grants of the Deutsche Forschungsgemeinschaft (CRC877, project A1) and the

German Cluster of Excellence 306 ‘Inflammation at Interfaces’. The Leeds Aneurysm Development Study was funded by the Garfield Weston Foundation and MB is supported by the British Heart Foundation.

Disclosures: Since October 2015, Daniel F. Freitag has been a full-time employee of Bayer AG, Germany.

References

1. Sakalihasan N, et al. Abdominal aortic aneurysm. *Lancet.* 2005;365:1577-1589.

2. Svensjo S, et al. Low prevalence of abdominal aortic aneurysm among 65-year-old Swedish men indicates a change in the epidemiology of the disease. *Circulation.* 2011;124:1118-1123.

3. Hager J, et al. Lower prevalence than expected when screening 70-year-old men for abdominal aortic aneurysm. *Eur J Vasc Endovasc Surg.* 2013;46:453-459.

4. Ulug P, et al. Meta-analysis of the current prevalence of screen-detected abdominal aortic aneurysm in women. *Br J Surg.* 2016;103:1097-1104.

5. Filardo G, et al. Surgery for small asymptomatic abdominal aortic aneurysms. *Cochrane Database Syst Rev.* 2015:Cd001835.

6. National Institute for Health and Care Excellence (NICE). Abdominal aortic aneurysm: diagnosis and management. NICE Guideline. Draft for consultation May 2018; 2018.

7. RESCAN Collaborators, *et al.* Surveillance intervals for small abdominal aortic aneurysms: a meta-analysis. *JAMA.* 2013;309:806-813.

8. Golledge J, et al. Current status of medical management for abdominal aortic aneurysm. *Atherosclerosis.* 2011;217:57-63.

9. Hellenthal FA, et al. Biomarkers of abdominal aortic aneurysm progression. Part 2: inflammation. *Nat Rev Cardiol.* 2009;6:543-552.

10. Nordon IM, et al. Pathophysiology and epidemiology of abdominal aortic aneurysms. *Nat Rev Cardiol.* 2011;8:92-102.

11. Raffort J, et al. Monocytes and macrophages in abdominal aortic aneurysm. *Nat Rev Cardiol.* 2017;14:457-471.

12. Harrison SC, et al. Interleukin-6 receptor pathways in abdominal aortic aneurysm. *European Heart Journal.* 2013;34:3707-3716.

American Heart Association.
Circulation: Genomic and Precision Medicine

13. Flondell-Site D, et al. Cytokines and systemic biomarkers are related to the size of abdominal aortic aneurysms. *Cytokine*. 2009;46:211-215.

14. Garbers C, et al. Interleukin-6: designing specific therapeutics for a complex cytokine. *Nat Rev Drug Discov*. 2018;17:395-412.

15. Rose-John S, et al. The role of IL-6 in host defence against infections: immunobiology and clinical implications. *Nat Rev Rheumatol*. 2017;13:399-409.

16. Chalaris A, et al. The soluble Interleukin 6 receptor: generation and role in inflammation and cancer. *Eur J Cell Biol*. 2011;90:484-494.

17. Sarwar N, et al. Interleukin-6 receptor pathways in coronary heart disease: a collaborative meta-analysis of 82 studies. *Lancet*. 2012;379:1205-1213.

18. Schnabel RB, et al. Large-scale candidate gene analysis in whites and African Americans identifies IL6R polymorphism in relation to atrial fibrillation: the National Heart, Lung, and Blood Institute's Candidate Gene Association Resource (CARe) project. *Circ Cardiovasc Genet*. 2011;4:557-564.

19. Eyre S, *et al*. High-density genetic mapping identifies new susceptibility loci for rheumatoid arthritis. *Nat Genet*. 2012;44:1336-1340.

20. Ferreira RC, et al. Functional IL6R 358Ala allele impairs classical IL-6 receptor signaling and influences risk of diverse inflammatory diseases. *PLoS Genet*. 2013;9:e1003444.

21. Ferreira MA, et al. Identification of IL6R and chromosome 11q13.5 as risk loci for asthma. *Lancet*. 2011;378:1006-1014.

22. Briso EM, et al. Cutting edge: soluble IL-6R is produced by IL-6R ectodomain shedding in activated CD4 T cells. *J Immunol*. 2008;180:7102-7106.

23. Garbers C, et al. The interleukin-6 receptor Asp358Ala single nucleotide polymorphism rs2228145 confers increased proteolytic conversion rates by ADAM proteases. *Biochim Biophys Acta*. 2014;1842:1485-1494.

24. Badger SA, et al. C-reactive protein (CRP) elevation in patients with abdominal aortic aneurysm is independent of the most important CRP genetic polymorphism. *J Vasc Surg*. 2009;49:178-184.

25. Parry DJ, et al. Haemostatic and fibrinolytic factors in men with a small abdominal aortic aneurysm. *Br J Surg*. 2009;96:870-877.

26. Salem MK, et al. Should Asian men be included in abdominal aortic aneurysm screening programmes? *Eur J Vasc Endovasc Surg*. 2009;38:748-749.

American Heart Association. Circulation: Genomic and Precision Medicine

27. Bown MJ, et al. Abdominal aortic aneurysm is associated with a variant in low-density lipoprotein receptor-related protein 1. *Am J Hum Genet.* 2011;89:619-627.

28. Brady AR, et al. Abdominal aortic aneurysm expansion: risk factors and time intervals for surveillance. *Circulation.* 2004;110:16-21.

29. Lindholt JS, et al. Screening for abdominal aortic aneurysms: single centre randomised controlled trial. *Bmj.* 2005;330:750.

30. McCaul KA, et al. Long-term Outcomes of the Western Australian Trial of Screening for Abdominal Aortic Aneurysms: Secondary Analysis of a Randomized Clinical Trial. *JAMA Intern Med.* 2016;176:1761-1767.

31. Bumdelger B, et al. Osteoprotegerin Prevents Development of Abdominal Aortic Aneurysms. *PLoS One.* 2016;11:e0147088.

32. Moran CS, et al. Association of osteoprotegerin with human abdominal aortic aneurysm progression. *Circulation.* 2005;111:3119-3125.

33. Golledge J, et al. Circulating markers of abdominal aortic aneurysm presence and progression. *Circulation.* 2008;118:2382-2392.

34. Barkhausen T, et al. Selective blockade of interleukin-6 trans-signaling improves survival in a murine polymicrobial sepsis model. *Crit Care Med.* 2011;39:1407-1413.

35. Zhang H, et al. IL-6 trans-signaling promotes pancreatitis-associated lung injury and lethality. *J Clin Invest.* 2013;123:1019-1031.

36. Gregory AP, et al. TNF receptor 1 genetic risk mirrors outcome of anti-TNF therapy in multiple sclerosis. *Nature.* 2012;488:508-511.

37. Lareyre F, et al. TGFβ (Transforming Growth Factor-β) Blockade Induces a Human-Like Disease in a Nondissecting Mouse Model of Abdominal Aortic Aneurysm. *Arteriosclerosis, Thrombosis, and Vascular Biology.* 2017.

38. Wang Y, et al. TGF-beta activity protects against inflammatory aortic aneurysm progression and complications in angiotensin II-infused mice. *J Clin Invest.* 2010;120:422-432.

39. Hu YJ, et al. Impact of Selection Bias on Estimation of Subsequent Event Risk. *Circ Cardiovasc Genet.* 2017;10.

40. Golledge J***, et al.*** Abdominal aortic aneurysm: pathogenesis and implications for management. *Arterioscler Thromb Vasc Biol.* 2006;26:2605-2613.

American Heart Association.
Circulation: Genomic and Precision Medicine

41. Furuya Y, et al. Interleukin-6 as a potential therapeutic target for pulmonary arterial hypertension. *Int J Rheumatol.* 2010;2010:720305.

42. Taniguchi K, et al. Tocilizumab is effective for pulmonary hypertension associated with multicentric Castleman's disease. *Int J Hematol.* 2009;90:99-102.

43. Smolen JS, et al. Effect of interleukin-6 receptor inhibition with tocilizumab in patients with rheumatoid arthritis (OPTION study): a double-blind, placebo-controlled, randomised trial. *Lancet.* 2008;371:987-997.

44. Surendran P, et al. Trans-ancestry meta-analyses identify rare and common variants associated with blood pressure and hypertension. *Nat Genet.* 2016;48:1151-1161.

45. Evans JM, et al. Increased incidence of aortic aneurysm and dissection in giant cell (temporal) arteritis. A population-based study. *Ann Intern Med.* 1995;122:502-507.

46. Shovman O, et al. Aortic aneurysm associated with rheumatoid arthritis: a population-based cross-sectional study. *Clin Rheumatol.* 2016;35:2657-2661.

47. Nozawa T, et al. Coronary-Artery Aneurysm in Tocilizumab-Treated Children with Kawasaki's Disease. *N Engl J Med.* 2017;377:1894-1896.

American Heart Association.
Circulation: Genomic and Precision Medicine

Doi: 10.1161/CIRCGEN.118.002413

Table 1. Baseline characteristics of the studies included in the human genetic analysis

	N	Average no. scans	Average baseline AAA size (mm)	Average years between first and last scan	Males (%)	Average age (years)	Current smokers (%)	Ever smokers (%)	Diabetes (%)
**Belfast[24]**	239	3	50	2.0	219 (92)	71.8	53 (22)	N/A	25 (10)
**Leeds[25]**	543	6	49	2.8	464 (85)	74.3	132 (24)	490 (90)	66 (12)
**Leicester[26]**	209	6	37	4.1	206 (99)	69.0	62 (30)	189 (90)	22 (11)
**Mayo**	530	2	40	4.2	448 (85)	70.1	N/A	471 (89)	128 (24)
**St George[27]**	79	2	56	1.4	71 (90)	77.2	16 (20)	51 (65)	4 (5)
**UKSAT[28]**	466	7	45	2.5	387 (83)	68.8	160 (34)	439 (94)	14 (3)
**Viborg[29]**	16	6	35	5.2	16 (100)	65.7	5 (31)	N/A	0 (0)
**Viva*[29]**	523	2	41	1.4	523 (100)	71.0	215 (41)	320 (61)	57 (11)
**WA[30]**	258	4	35	4.8	258 (100)	72.7	46 (18)	221 (86)	27 (10)
**TOTAL**	**2,863**	**4**	**43**	**3.1**	**2,592 (91)**	**71.7**	**689 (24)**	**2,181 (76)**	**343 (12)**

Notes: (1) Abbreviations: AAA= Abdominal Aortic Aneurysm; WA=Western Australia. (2) Missing data: sex=1(0.1%), age=3(0.1%), current smoking=566(20%), ever smoking=456 (16%), diabetes=98 (3%). *170 participants in Viva do not have data for date of measurement.

Circulation: Genomic and Precision Medicine

Table 2. Comparison of results for the association between IL-6R and abdominal aortic aneurysm from human genetic analysis and mouse experimental models

Model	Inhibition of IL-6 signalling	Effect on AAA growth/ survival	p-value of effect	Other cardiovascular markers (p<0.05)
**Humans stratified for *IL6R*-Asp358Ala**	Minor allele associated with dampening of classical signalling. Effects on trans-signalling not clear.	Non-significant change in AAA growth (0.06mm/year)*	0.136	Increased IL-6, sIL-6R, CXCL10, CXCL11, IL1α and CD6;<br>Reduced MMP3, OPG, IGFBP1, TIMP4
**Angiotensin II + anti-TGFβ**	Both classical and trans-signalling	No difference in survival	1.000	Increased IL-6;<br>Reduced SAA, IL-2, IL-5, CXCL1 and SBP
**mouse model**	Only trans-signalling	Improved survival	0.040	Increased IL-5;<br>Reduced TNFα
**Elastase + anti-TGFβ**	Both classical and trans-signalling	Decreased survival	0.029	Increased recruitment of $CD3^{+}$ T cells
**mouse model**	Only trans-signalling	Improved survival	0.024	Increased collagen content of the arterial wall

*Effect indicated per copy of the IL6-R minor allele.

American Heart Association. Circulation: Genomic and Precision Medicine

Doi: 10.1161/CIRCGEN.118.002413

Figure Legends:

Figure 1: Sex- and age-adjusted change in abdominal aortic aneurysm growth rate (mm/year) per copy of the IL-6 minor allele

Figure 2: Anti-IL6-R prevents angiotensin II induced hypertension but does not protect against aortic rupture induced by angiotensin II and anti-TGFβ infusion. Mice were treated with anti-IL6-R or isotype control (n=22 mice/group) starting one week before angiotensin II and anti-TGFβ infusion. A – Plasma concentration of cytokines at day 0 (before angiotensin II and anti-TGFβ infusion) and day 7 to 14. *$p<0.05$ isotype vs anti-IL6-R; **$p<0.01$ isotype vs anti-IL-6R; 2-way ANOVA followed by uncorrected fisher's test. B – Plasma concentration of serum amyloid A (SAA) at day 0 (before angiotensin II and anti-TGFβ infusion) and day 7 to 14. ***$p<0.05$ isotype vs anti-IL6-R; 2-way ANOVA followed by uncorrected fisher's test. C - Systolic blood pressure measurement using tail cuff at day 0 and day 7 after angiotensin II and anti-TGFβ infusion. ***$p<0.001$ isotype vs anti-IL-6R; 2-way ANOVA followed by uncorrected fisher's test. D - Survival curves of mice after angiotensin II and anti-TGFβ infusion. All data for the generation of the graphs shown in Figure 2 were generated in two independent experiments and then pooled together.

Figure 3: Selective blockage of the IL-6 trans-signalling pathway using sgp130 in the angiotensin II + anti-TGFβ model reduces aortic rupture. Mice were treated with sgp130-Fc or human IgG1 Fc (Hum-Fc) (n=10 mice/group) starting one week before angiotensin II and anti-TGFβ infusion. A - Plasma concentration of cytokines at day 0 (before angiotensin II and anti-

American Heart Association
Circulation: Genomic and Precision Medicine

TGFβ infusion) and day 7. *p<0.05 Hum-Fc vs sgp130; ***p<0.001 Hum-Fc vs sgp130; 2-way ANOVA followed by uncorrected fisher's test. B - Plasma concentration of serum amyloid A (SAA) at day 0 (before angiotensin II and anti-TGFβ infusion) and day 7. C - Systolic blood pressure measurement using tail cuff at day 0 and day 7 after angiotensin II and anti-TGFβ infusion. D - Survival curves of mice after angiotensin II and anti-TGFβ infusion. *p<0.05 Hum-Fc vs sgp130; Gehan-Breslow-Wilcoxon test. All data for the generation of the graphs shown in Figure 3 were generated in one independent experiment.

Figure 4: Blockage of the IL-6 pathway using anti-IL-6R in the elastase + anti-TGFβ model enhances T cell infiltration and rupture of the aorta. Mice were treated with anti-IL-6R or isotype control (n=12 mice/group) starting one week before the application of elastase and the infusion anti-TGFβ. A - Survival curves of mice after the application of elastase and the infusion anti-TGFβ. *p<0.05 isotype vs anti-IL6-R; Gehan-Breslow-Wilcoxon test. B - Representative macroscopic pictures of abdominal aortic aneurysms from mice treated with elastase and anti-TGFβ and isotope or anti-IL6-R, at day 16. Note that the aneurysm from the isotype treated mouse was not ruptured. C - Analysis of the aortic diameter (µm) based on the perimeter obtained from aortic cross sections. D - Quantification and representative images of collagen content of the aortic wall analysed using Sirius Red staining under polarized light. E, F - Quantification and representative images of MPO (D) and CD3 (E) immunofluorescent stainings on aortic cross section. *p<0.05 isotype vs anti-IL-6R; Mann-Whitney test. All data for the generation of the graphs shown in Figure 4 were generated in one independent experiment.

American Heart Association
Circulation: Genomic and Precision Medicine

Figure 5: Selective blockage of the IL-6 trans-signalling pathway using sgp130 in the elastase + anti-TGFβ model increased collagen deposition and prevent aortic rupture. Notes: Mice were treated with sgp130 or Hum-Fc (n=12 mice/group) starting on the day of the application of elastase and the infusion anti-TGFβ. A - Survival curves of mice after the application of elastase and the infusion anti-TGFβ. *$p<0.05$ Hum-Fc vs sgp130; Gehan-Breslow-Wilcoxon test. B - Representative macroscopic pictures of abdominal aortic aneurysms from mice treated with elastase and anti-TGFβ and isotope or anti-IL6-R, at day 16. Note that the aneurysm from the Hum-Fc treated mouse was ruptured. C - Analysis of the aortic diameter (μm) based on the perimeter obtained from aortic cross sections. D - Quantification and representative images of collagen content of the aortic wall analysed using Sirius Red staining under polarized light. *$p<0.05$ Hum-Fc vs sgp130; Mann-Whitney test. E, F - Quantification and representative images of Ly6G (D) and CD3 (E) immunofluorescent stainings on aortic cross section. All data for the generation of the graphs shown in Figure 5 were generated in one independent experiment.

American Heart
Circulation: Genomic and Precision Medicine

Figure 6: Overview of the IL-6 classical and trans-signalling pathways and their potential role in AAA growth. Notes: A - In “classical” IL-6 signalling (left), the binding of the cytokine IL-6 to the membrane-bound IL-6 receptor (mIL-6R) leads to the dimerization of its co-receptor gp130, and subsequently, triggers downstream signalling in a restricted subset of cells. In IL-6 trans-signalling (right), IL-6 forms a complex with soluble IL-6 receptor (sIL-6R) that can stimulate cells expressing gp130 even in the absence of mIL-6R. The minor allele of a functional variant in the *IL6R* gene, Asp358Ala (rs2228145 A>C) results in more efficient proteolytic cleavage of mIL-6R, thereby reducing levels of mIL-6R and classical IL-6 signalling but potentially increasing trans-signalling. B - In mouse models of AAA, the blockage of both the classical and

trans-signalling pathways with anti-IL6-R (i.e. MR16-1, the animal-equivalent of tocilizumab) did not have a conclusive effect on the time to aneurysm rupture. C - Specific blockage of the trans-signalling pathway with sgp130 resulted in improved survival rates in mouse models of AAA.

American Heart Association
Circulation: Genomic and Precision Medicine

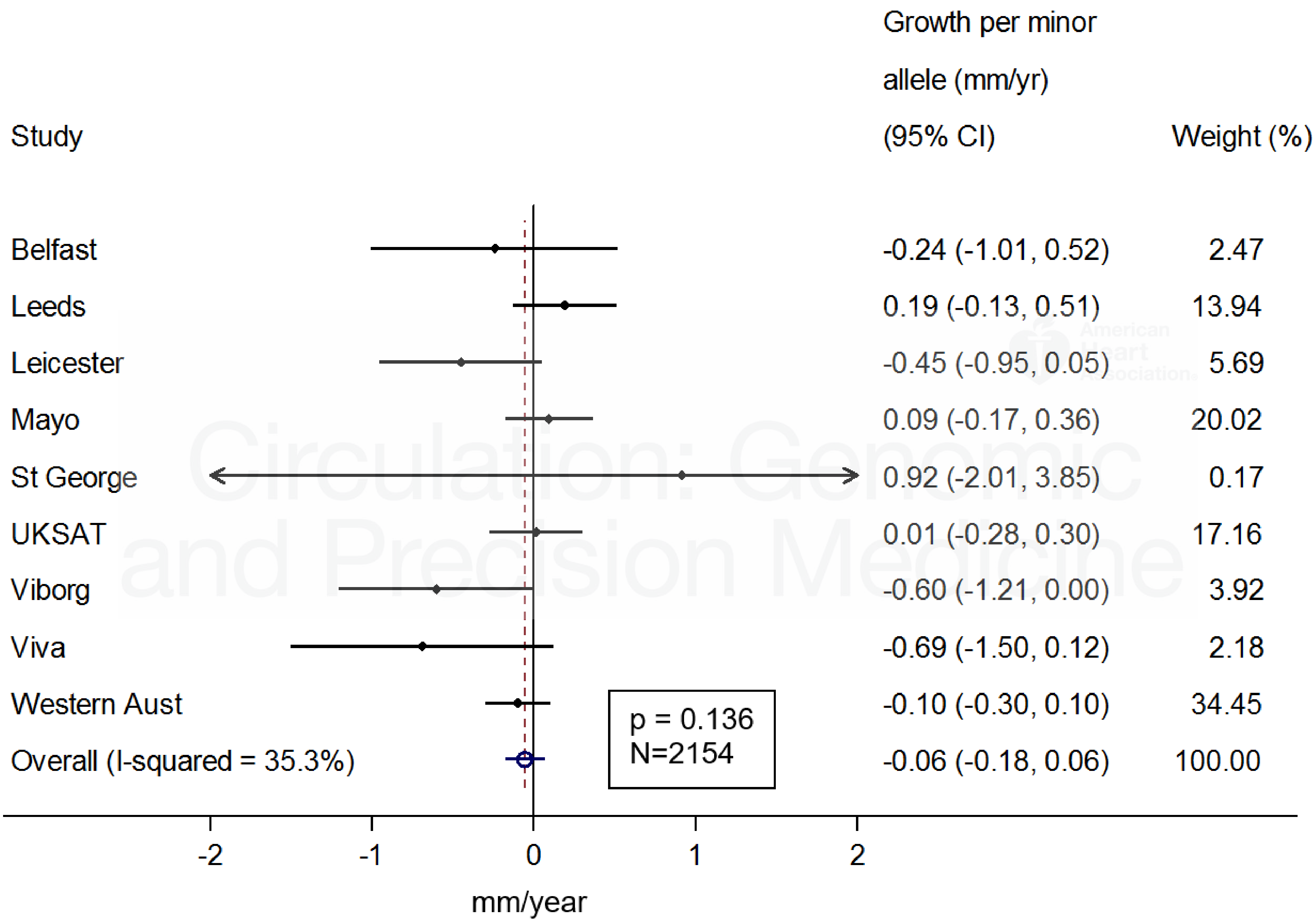

Circulation: Genomic and Precision Medicine

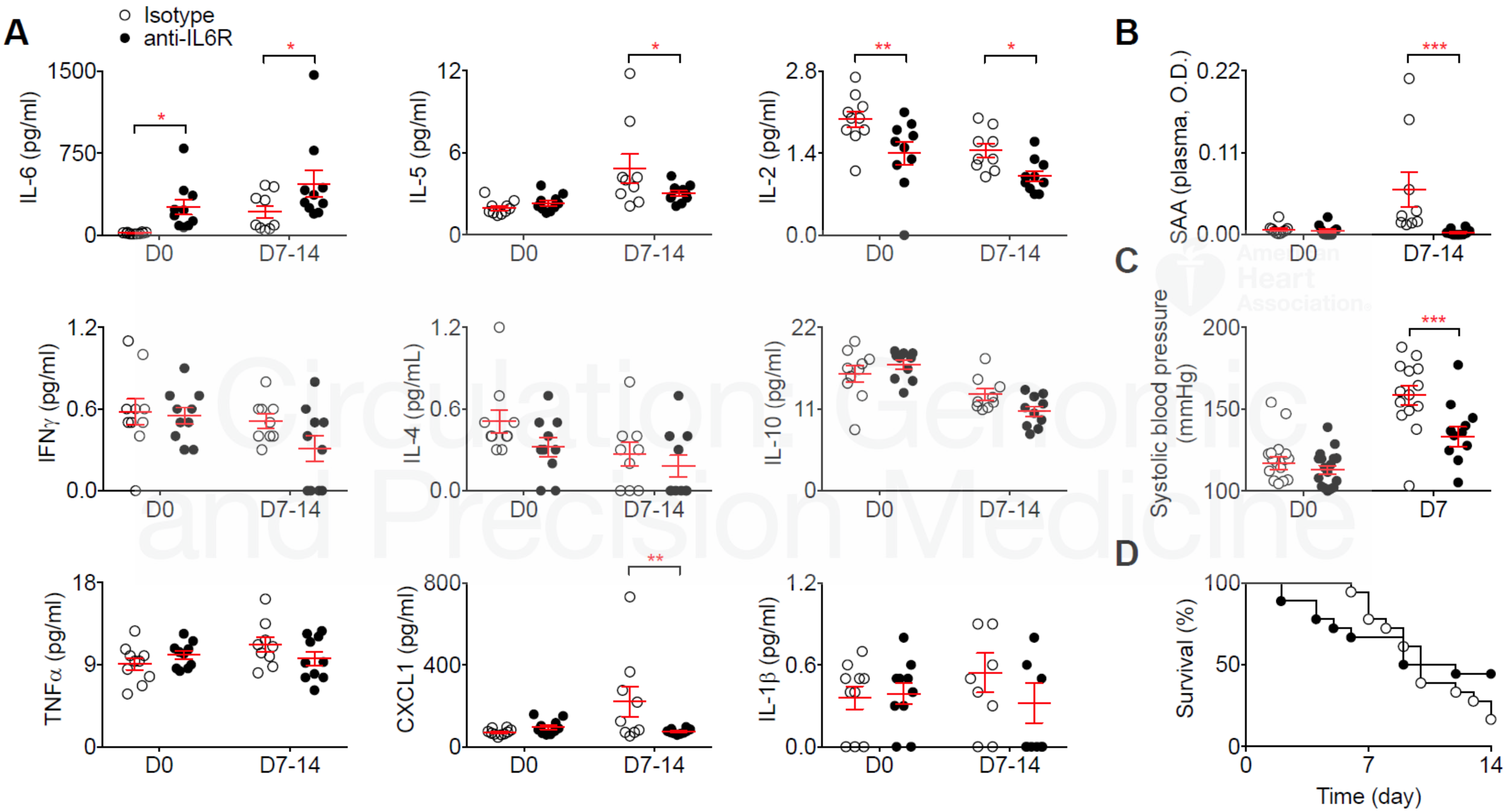

Circulation: Genomic and Precision Medicine
American Heart Association

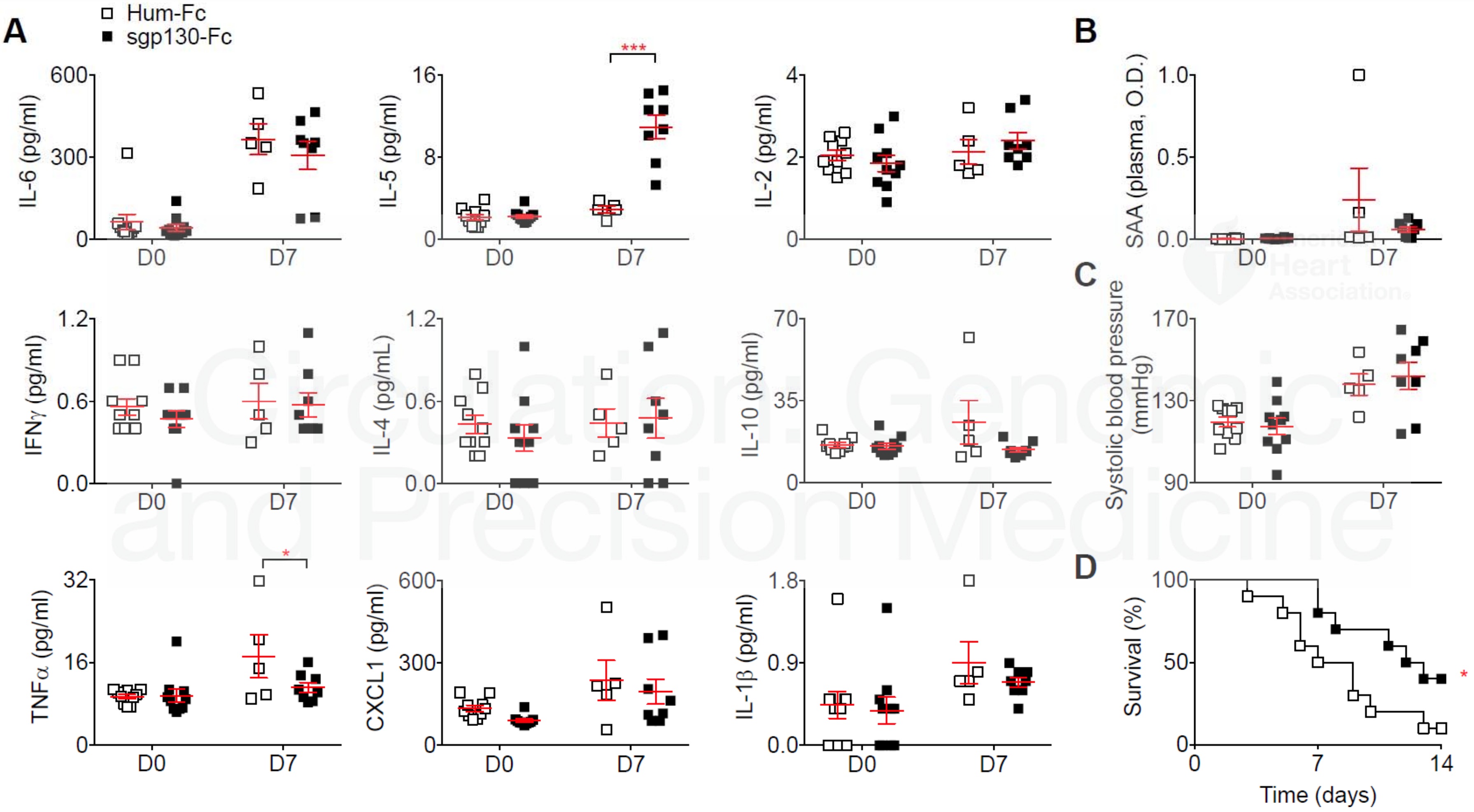

American Heart Association.
Circulation: Genomic and Precision Medicine

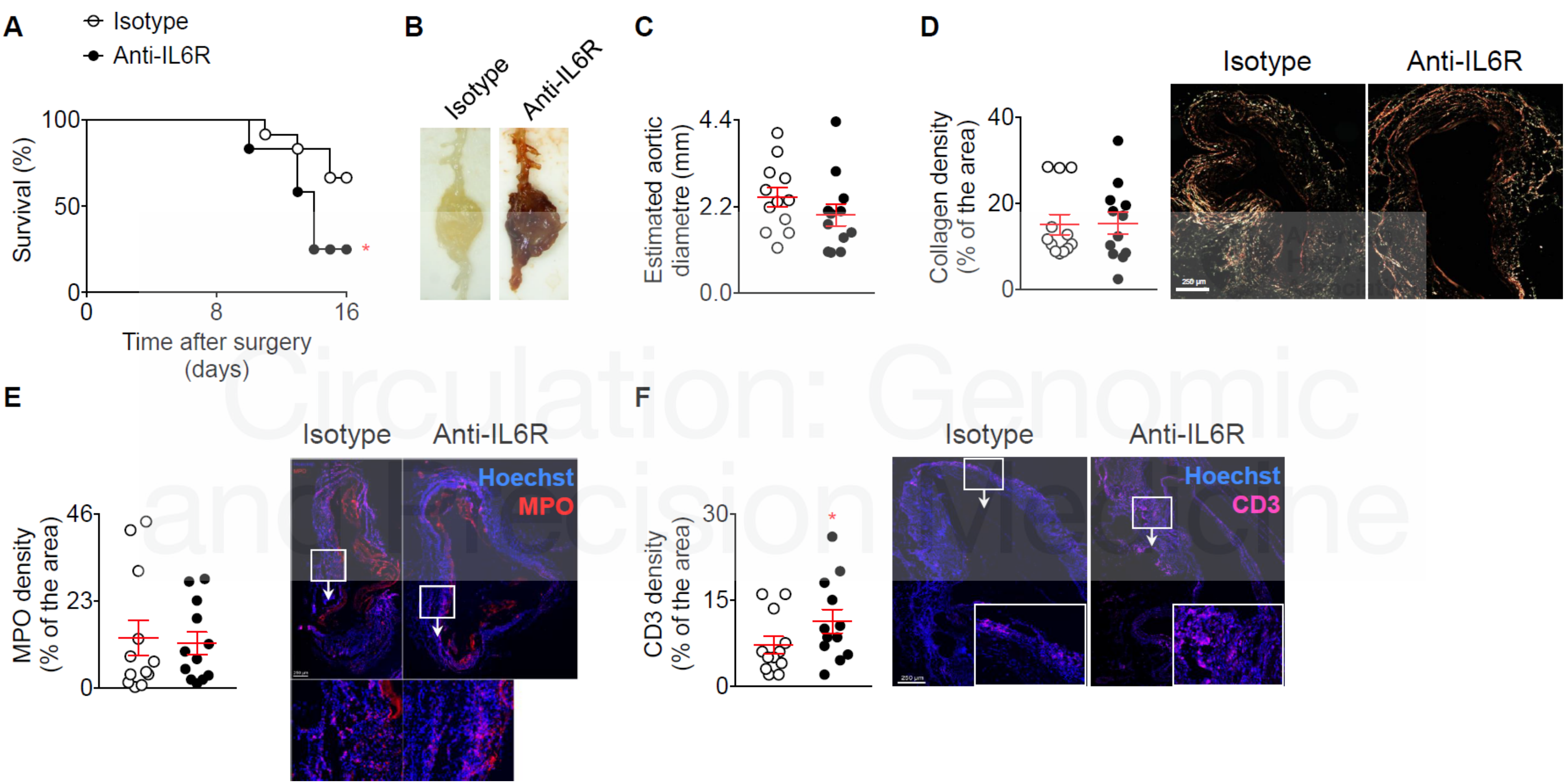

Circulation: Genomic and Precision Medicine

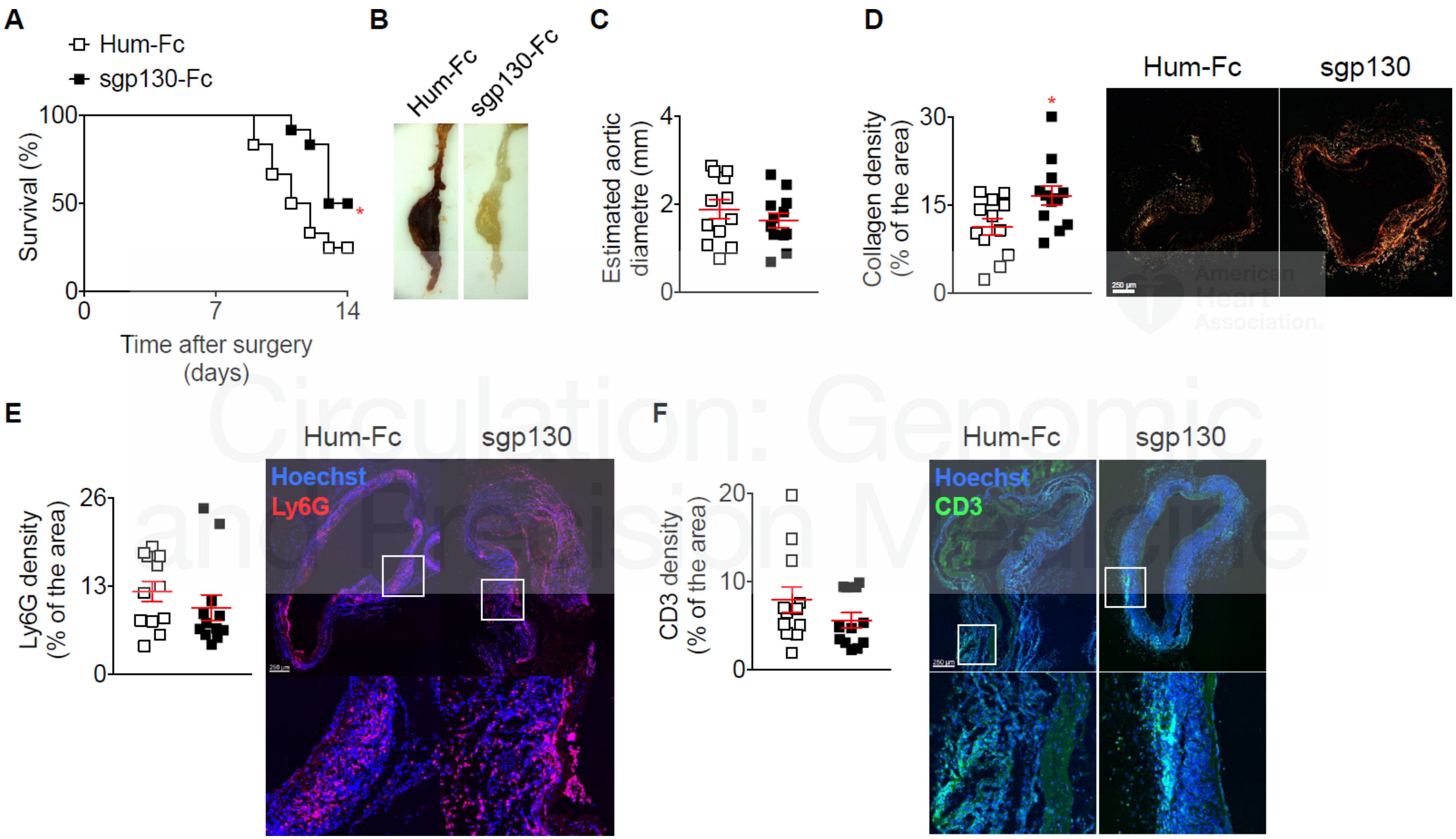

American Heart Association.
Circulation: Genomic and Precision Medicine

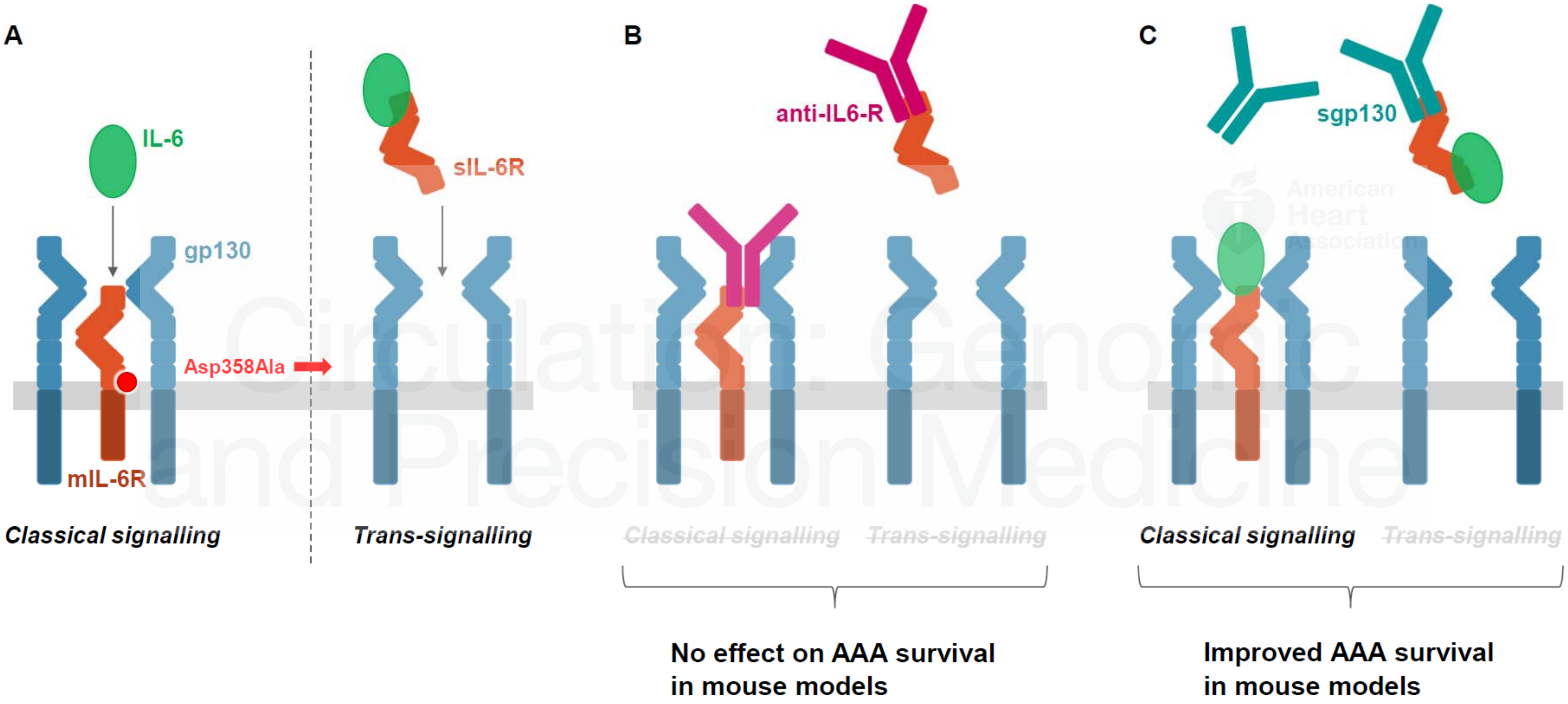

Circulation: Genomic and Precision Medicine
American Heart Association
